# Unveiling the Microbial Signatures of Arabica Coffee Cherries: Insights into Ripeness Specific Diversity, Functional Traits, and Implications for Quality and Safety

**DOI:** 10.3390/foods14040614

**Published:** 2025-02-12

**Authors:** Gabriela N. Tenea, Victor Cifuentes, Pamela Reyes, Marcelo Cevallos-Vallejos

**Affiliations:** Biofood and Nutraceutics Research and Development Group, Faculty of Engineering in Agricultural and Environmental Sciences, Universidad Técnica del Norte, Ibarra 100150, Ecuador

**Keywords:** coffee, microbial diversity, lactic acid bacteria, antibiotic resistant genes, biocide, metal-resistant genes

## Abstract

Arabica coffee, one of the most valuable crop commodities, harbors diverse microbial communities with unique genetic and functional traits that influence bean safety and final coffee quality. In Ecuador, coffee production faces challenges due to the spread of pathogenic organisms across cultivars, leading to reduced yields and compromised quality. This study employed a shotgun metagenomic approach to characterize the indigenous microbial diversity present in the cell biomass of fermented coffee cherries from three *Coffea arabica* varieties: Typica (Group A), Yellow Caturra (Group B), and Red Caturra (Group C), originating from the Intag Valley in northern Ecuador, at two ripe stages: green (immature fruits) and ripe (red/yellow mature fruits). Gene prediction and functional annotation were performed using multiple databases, including EggNOG, COG, KEGG, CAZy, CARD, and BacMet, to explore the potential impact of microbial communities on bean quality and safety. Metagenomic sequencing generated over 416 million high-quality reads, averaging 66 million clean reads per sample and yielding a total of 47 Gbps of data. Analysis revealed distinct differences in species abundance based on the coffee variety and ripening stage. A total of 799,658 protein-coding sequences (CDSs) were predicted, of which 205,937 genes were annotated with EggNOG, 181,723 with COG, 155,220 with KEGG, and 10,473 with CAZy. Additionally, 432 antibiotic resistance genes (ARGs) were identified using CARD, and 8974 biocide and metal resistance genes (BMRGs) were annotated with BacMet. Immature cherries exhibited enriched pathways associated with resistance to antibiotics such as fluoroquinolones, penams, rifamycin, macrolides, carbapenems, and cephalosporins. The abundance of these pathways varied with the ripening stage and variety. Furthermore, green cherries showed a significant increase in BMRGs associated with resistance to substances including hydrochloric acid, copper, nickel, hydrogen peroxide, arsenic, and zinc. Among mature cherries, Typica and Red Caturra shared similar profiles, while Yellow Caturra displayed a divergent microbial and functional profile. These study findings emphasize the interplay between microbial diversity, ripening stages, and coffee varieties, providing a foundation for innovative approaches to enhance coffee quality through microbiome management.

## 1. Introduction

One of the most significant and valuable commodities traded internationally is Arabica coffee (*Coffea arabica* L.), which accounts for about 70% of all coffee consumed worldwide [1]. Ecuador is one of the most biodiverse countries in the world, thanks to its strategic geographical location and favorable climate, which support a wide variety of crops [2]. Coffee cultivation in Ecuador primarily takes place in agroforestry polyculture systems, which not only enhance plantation sustainability but also contribute to biodiversity conservation [3]. In Ecuadorian coffee plantations, the most common tree species include fruit trees such as *Musa paradisiaca* (banana), *Carica papaya* (papaya), *Citrus limon* (lemon), *Psidium guajava* (guava), *Inga edulis* (ice cream bean), and *Theobroma cacao* (cacao), along with timber trees like *Cordia alliodora* and *Ochroma* spp. [3]. According to ANECAFE, Ecuador’s coffee production in 2022 reached 7.97 thousand metric tons across 364.000 hectares of cultivated land [4]. However, in 2023, national coffee production declined, unable to meet the steadily growing local demand, which has been increasing by 5% to 10% annually. Despite its many advantages, the coffee sector faces significant challenges, including the impacts of climate change, which have accelerated the spread of pathogenic organisms among coffee plants [5]. Additionally, production is constrained by inadequate storage facilities and declining market prices.

The Intag Valley, located in Imbabura Province at an average elevation of 1900 m above sea level, plays a crucial role as a protective buffer zone for the Cotacachi-Cayapas Ecological Reserve and the ecologically significant Ecuadorian Chocó bioregion, renowned for its exceptional biodiversity. This region plays a critical role in preserving Ecuador’s biodiversity, including many endangered species. Coffee cultivation plays a crucial economic and social role, providing significant income and employment for thousands of families across various communities and ethnic groups [6]. This approach promotes an eco-friendly production model with stable pricing, as it integrates the cultivation and sale of diverse products such as fruits, livestock, and timber, all within a single farm system. Additionally, this model enhances the food sovereignty and overall resilience of the farming communities. Among coffee varieties, *C. arabica* var. Yellow Caturra, *C. arabica* var. Red Caturra, and *C. arabica* var. Typica are cultivated in the region (http://aacri.com). Caturra Red (Caturra Rojo) and Caturra Yellow (Caturra Amarillo) share the same genetic base derived from the Bourbon variety within the Typica-Bourbon (Brazil) genetic group of *Coffea arabica*; they have diverged slightly due to genetic mutations in fruit color. Both share a single-gene mutation causing dwarfism, affecting plant height while retaining Bourbon’s key traits (https://varieties.worldcoffeeresearch.org/es/variedades/caturra, accessed on 5 February 2025). The primary genetic difference lies in a mutation in the anthocyanin biosynthesis pathway, which gives Caturra Yellow its yellow cherries instead of the red ones found in Caturra Red, without significantly altering agronomic or cup quality characteristics. The Red Caturra begins tasting sweeter than its Yellow counterpart. The three main flavors are chocolate, berries, and grape. The Yellow Caturra is a “rare” varietal that tends to ripen faster than the Red Caturra and is more acid and almost lime-like. The Typica variety is mild, sweet, and pleasant. Chocolate and nut notes are prominent, with hints of spice to balance them. The mild, well-balanced acidity of Typica conveys dried and dark stone fruits like black cherry and plum.

The quality of coffee is determined by the makeup of the coffee bean. In addition, the coffee genotype, environmental conditions, and altitude might influence the coffee quality [7]. Previous research has explored the diversity of microorganisms associated with coffee cherries, particularly focusing on beneficial microorganisms such as lactic acid bacteria [8]. Gaining a deeper understanding of the microbial diversity linked to coffee cherries, along with identifying microbial genes related to metabolic functions or metabolites influencing flavor and texture, is crucial for enhancing coffee production and genetic improvement [9]. Furthermore, the microbiome of coffee beans plays a critical role in augmenting the coffee’s flavor profile by producing aromatic compounds through spontaneous fermentation [10,11,12].

The metagenomic approach is an effective tool for investigating the ecology and metabolic profiles of microorganisms, as well as for discovering new biomolecules such as proteins and enzymes [13]. A recent metagenomic study conducted in Ecuador during the fermentation processes of coffee (*C. arabica* var. Typica) demonstrated changes in the gene repertoire and temporal alterations to the coffee microbiome in connection with the dynamic fluctuations of the fermenting mass [14]. On the other hand, enhancing flavor quality requires evaluating fermentation ability and isolating microorganisms. Secondary metabolites and volatile compounds linked to flavors and aromas are produced by the metabolic pathways of microorganisms [15]. Nonetheless, the quality of the final green coffee beans depends on the quality and safety of the cherries as well as the postharvest processing steps. Additionally, farming practices and geographic location can impact the microbiome [16]. Moreover, there is growing evidence that metal contamination is an environmental factor contributing to antibiotic persistence and resistance within the microbiome [17]. To date, several studies have focused on the postharvest processing steps of coffee beans to identify the microbiota linked to the fermentation stage of the processing and to profile the chemical composition of the green coffee beans and/or the process of bean germination; however, limited knowledge still exists regarding the microbial diversity and their metabolites function in the transition from the immature to the mature ripe stage of coffee cherries.

The current work carried out a shotgun metagenomic analysis on six cherry pool samples harvested from three local varieties at two ripe stages (green: red, green: yellow) during the fermentation process under laboratory conditions. Specifically, the composition and diversity of indigenous microorganisms were evaluated. Additionally, we focused on the gene prediction and annotation of the metagenomic reads or contigs to mine metabolic pathways (EggNOG, COG, KEGG, CAZy), antibiotic-resistant genes (ARGs), and biocide- and metal-resistant genes (BMRGs) to ascertain the possible functional role of the cherries’ quality. Understanding microbial dynamics in coffee cherries offers opportunities to optimize fermentation processes by selecting beneficial microbial strains that enhance coffee quality while reducing undesirable microbial activity.

## 2. Materials and Methods

### 2.1. Sample Collection, Processing, and on Site Natural Fermentation

**Figure 1:**
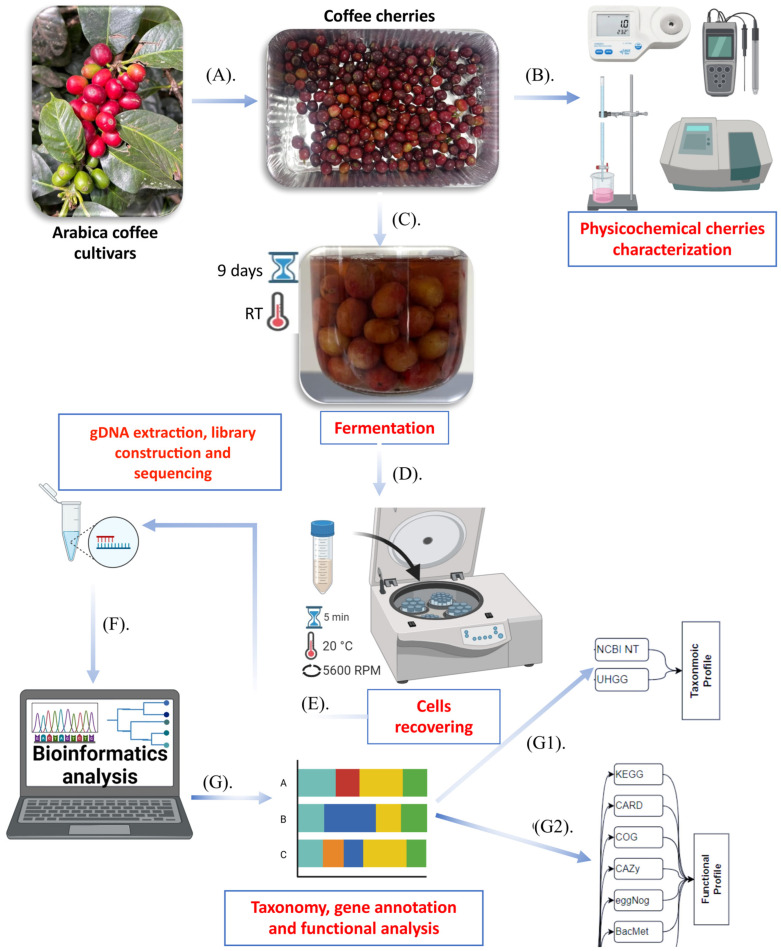
Overview of the fruits; processing and analysis. (**A**) coffee cherries at different ripe stage recollections; (**B**) physicochemical analysis of cherries; (**C**) laboratory wet fermentation; (**D**) cells recollection; (**E**) cell recovering and gDNA extraction, library construction and sequencing; (**F**) bioinformatics analyses; (**G1**) cell microbiome taxonomy; (**G2**) gene annotation, functional analysis.

In this study, cherries of three *Coffea arabica* L. varieties: Typica (annotation code: group A), Yellow Caturra (annotation code: group B), and Red Caturra (annotation code: group C), at two ripe stages: immature green (samples A1, C1, B1), mature red (samples A2, C2), and mature yellow (sample B2), a total of six pool samples (500 g cherries per sample), were obtained from a coffee farming setting of the Intag Valley (geographical coordinates 0°21′0″ N, 78°44′0″ W) parish of Peñaherrera. The soil in this region is volcanic. The fruits were collected in sterile bags as follows: two ripeness stages × three varieties × two colors, transported to the laboratory, and processed. The fruits were thoroughly washed and allowed to undergo natural fermentation in sterile flasks containing 100 mL of water at room temperature for 9 days. The cell biomass (corresponding to six metagenomes) was recovered in 1 × PBS by centrifugation of fermented liquid at 8000× *g* for 5 min and stored at 4 °C before analysis. An overview of the workflow process is shown in Figure 1.

### 2.2. Shotgun Metagenomic Sequencing 

#### 2.2.1. DNA Extraction and Library Construction

Genomic DNA was isolated from the cells recollected from the fermentation step using a commercial column-based ZymoBIOMICS DNA miniprep kit following the instructions of the manufacturer (#4300, Zymo Research, Irvine, CA, USA). Before constructing the DNA library, the concentration (≥12.5 μg/μL), integrity (main peak > 20 kb), and purity (no protein, RNA, and other contamination) were checked using a Qubit Fluorometer following the instructions of the manufacturer. In brief, the library workflow consisted of (1) sample disruption: genomic DNA (1 μg) was disrupted using an ultrasonicator (Covaris 2, PerkinElmer CO, Walthman, MA, USA); (2) Fragment size selection was performed on the samples using magnetic beads to concentrate the DNA fragments in the range of 200–400 bp; (3) End repair, A-tailing, and adapter ligation: to repair the ends of double-stranded cDNA, a reaction mixture was prepared. This step also involves adding an “A” base to the 3′ end. An adapter ligation reaction mixture was used to ligate the adapters to the DNA (4) PCR amplification and product recovery: purification and the recovery of the amplified ligated products were performed using magnetic beads. (5) Circularization of products: after the PCR product was denatured into a single chain, the cyclization reaction system was prepared, and the single-chain ring product was obtained by fully mixing the reaction at the right temperature for a certain time. After digesting the uncyclized linear DNA molecule, the final library was obtained. (6) Library quality control: the cyclization product was used to detect the concentration before sequencing.

#### 2.2.2. Sequencing and Data Processing

Metagenome sequencing was performed on the DNBSEQ-T7 platform (Complete Genomics, San Jose, CA, USA) with a read length of 150 bp following the manufacturer’s instructions (custom by demand service). Data processing and functional annotation were performed following a pipeline workflow developed by the BGI manufacturer (Genomic Groups, Shenzhen, China). In brief, after the sequencing is performed, preprocessing is necessary to obtain valid data (clean data) that can be used for subsequent analysis. This includes removing low-quality reads, reads with excessive N bases, and non-genomic sequences, primarily those introduced during library preparation (adapters, primers, and indexes). The quality control of sequencing reads and removal of host reads was performed using a SOAPnuke [18]. Further, Bowtie2  was used to align the sequences to the host genome, remove the aligned sequences (host), and generate clean data [19]. This step involved the removal of 10% or more of uncertain bases (N bases); the removal of reads containing sequencing adapter sequences (regions with 15 or more consecutive bases aligned to adapter sequences); and the removal of reads with over 50% low-quality bases (bases with a quality score below 20).

#### 2.2.3. Metagenome De Novo Assembly, Gene Prediction, and Functional Annotation

MEGAHIT was used to perform k-mer-based assembly of the quality-controlled and host-removed sequences to generate contigs [20]. From the assembly results, fragments shorter than 300 bp were removed to facilitate further statistical analysis and gene prediction. The assembly results were further assessed for completeness (N50, N90, min length) and continuity (assembly length, max length). Next, MetaGeneMark software was employed to predict gene sequences within the contigs [21]. De novo prediction depends on distinct feature information between coding and non-coding regions and is based on sequence features that are provided. Building probability models to separate coding from non-coding regions was explained statistically. Both known and unknown genes were predicted. Further, the CD-HIT was used to remove redundancy (deduplication) from the obtained genes [22]. The longest sequence was automatically divided into the first class and served as the representative sequence of the first class. Then, the remaining sequences were compared with representative sequences discovered earlier. Based on sequence similarity (typically setting an identity threshold of 95% and a coverage threshold of 90%), each sequence was assigned to one class or became the representative sequence of a new cluster. This process was repeated for all sequences to complete the clustering process. Then, Salmon was employed to quantify the relative abundance of each gene [23]. Finally, DIAMOND software [24] was used to align non-redundant genes to various databases for functional annotation. The Comprehensive Antibiotic Resistance Database CARD v. 3.0.9 [25] and Antibacterial Biocide and Metal Resistance Genes Database BacMet v. 20180311 [26] were used to mine the antibiotic and metal gene resistance. For functional annotation, the Evolutionary genealogy of genes: Non-supervised Orthologous Groups: EggNOG v. 5.0 [27], the Kyoto Encyclopedia of Genes and Genomes: KEGG v. 101 [28], the Clusters of Orthologous Groups: COG v. 20201125, [29], and Carbohydrate-Active enZYmes Database: CAZy v. 20211013 [30] and Swiss-Prot release-2021_04 [31] were used.

### 2.3. Taxonomic Annotation and Data Analysis

To extract the plant genome annotation, the genome assembly *Coffea arabica* ET-39 HiFi was used (https://www.ncbi.nlm.nih.gov/datasets/genome/GCA_036785885.1/, accessed on 10 May 2024). Kraken2 [32] and a custom database UHGG (filtered NCBI NT database) [33] were used to align sequences from the sample to calculate the number of sequences belonging to different species. Then, Bracken2 (Bayesian Re-estimation of Abundance with KrakEN) was used to estimate the actual abundance of species in the sample, completing the taxonomic annotation [34].

### 2.4. Visualization and Statistical Significance Tests

Based on gene abundance tables, species abundance tables, and functional abundance tables, visualizations and analyses of gene, species, and functional distribution were performed. Intra-sample diversity (alpha diversity) was determined by the Shannon diversity index [35], Simpson dominance index (species dominance in ecological surveys) [36] and Chao 1 (richness: number of species in a community) [37] indexes. Alpha diversity was evaluated using a nonparametric Kruskal–Wallis test or a one-way ANOVA on ranks to determine whether the samples originated from the same distribution [38]. The null hypothesis for these tests assumes that the mean ranks of the groups are equal. Beta diversity, which assesses inter-sample diversity [39,40], was analyzed to identify differences between samples using permutational multivariate analysis of variance (PERMANOVA) based on Bray–Curtis dissimilarity scores and Euclidean distance matrices [41]. Additionally, ANOSIM (analysis of similarities) was employed to test the hypothesis that there are no differences between two or more groups of samples, relying on permutation testing of similarities within and between groups. Further analyses included heatmaps and Partial Least Squares Discriminant Analysis (PLS-DA) [42]. conducted using internal scripts to identify differences in species across the samples. The R test statistic was applied to assess the null hypothesis that no differences exist between the groups [43]. Functional differences between samples were explored through KEGG pathway enrichment analysis using the report score method.

### 2.5. Physicochemical Parameters Estimation

The pH of the cherries was measured using juice extracted by crushing the fruits in a mortar, while the pH of the ferment was assessed in the liquid juice after the fermentation using an electrode immersion pH meter (S210, Mettler Toledo, Columbus, OH, USA). Total soluble solids (TSSs) content was determined using a digital refractometer [44]. Using phenolphthalein as an indicator, the total titratable acidity (TTA) was measured by titrating 25 mL of pulp juice obtained with 0.1 N NaOH [44]. Results were expressed as a percentage of citric acid per milliliter of cherry juice and lactic acid per milliliter of fermented juice.

## 3. Results and Discussion

### 3.1. Metagenome Sequencing General Data

In this study, metagenomic sequencing generated over 416 million quality reads, averaging 66 million high-quality reads per sample, totaling 47 Gbps of data. Metagenome data was extracted by aligning the sequences to *Coffea arabica* ET-39 HiFi, achieving a clean data rate of 96.31%, with only 0.15% aligning to the host genome. Filtered raw data and quality control results are detailed in Table S1. Previous studies, such as Pothakos et al. [14], which analyzed six fermented *C. arabica* samples using *C. canephora* (Robusta) as the reference genome, may have faced limitations in accurately removing host sequences. Here, we present the first comprehensive functional analysis of the microbiome in naturally fermented coffee cherries, leveraging high-quality assembly libraries (300,797 contigs). The contigs’ average length, N50, and N90 values varied across samples, with the highest N50–N90 values reflecting superior assembly quality, facilitating accurate gene prediction and annotation (Table 1).

### 3.2. Bacterial Community Composition

**Figure 2:**
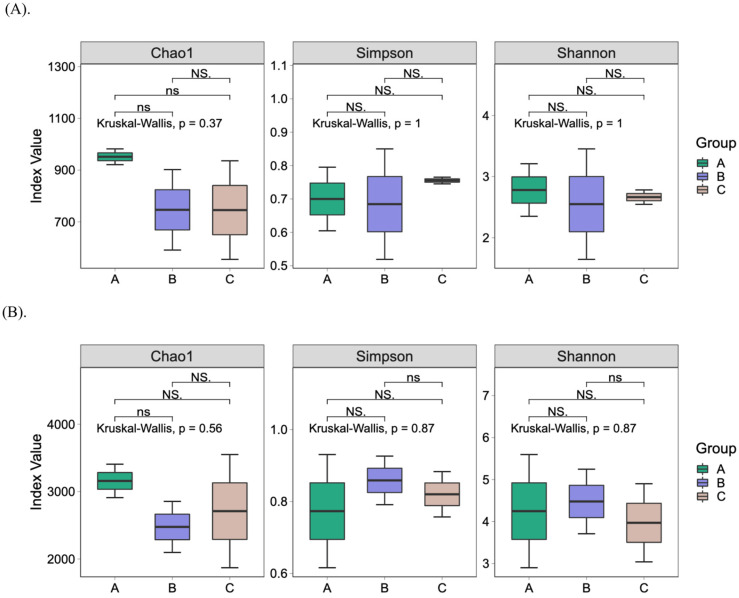
Alpha diversity results. Chao 1 (richness), Simpson (dominance) and Shannon (diversity) indices values for each group at (**A**) genus, and (**B**) species levels. Legend: group A: *C. arabica* L. var. Typica; group B: *C. arabica* L. var. Yellow Caturra; group C: *C. arabica* L. var. Red Caturra; NS: no significant differences.

The bacterial community composition of fermented cherries at unripe and ripe stages was evaluated. We used a 9-day fermentation period, longer than typical, as we worked with green cherries not intended for immediate coffee production. Green cherries, being immature, may require more time to break down certain compounds and promote microbial activity. The extended fermentation allowed for better microbial profiling [45]. Additionally, the geographical location and humid environment in the Intag Valley naturally support a longer fermentation process, as reported by local cultivators. Chao1 indices indicate the richness of species in the sample, whereas Shannon and Simpson indices show the diversity of the community, including species diversity and dominance (Table S2). Figure 2A displays the species diversity indices among the groups. Based on the Kruskal–Wallis statistical analysis, there were no statistically significant differences in diversity and dominance indexes between the groups. Even though group A had a higher richness index determined at the genus level, this was not significant (*p* = 0.37). We observed a higher Shannon index in the samples A1, C1, and B2 (Table S2). No statistical difference (*p* = 0.87) was seen when comparing all samples at the species level indicating that the fruits shared similar bacterial diversity (Figure 2B).

The correlation analysis (Pearson) between the diversity indexes revealed a strong positive relationship between the Shannon and Simpson indices (r = 0.93), indicating that as the Shannon diversity increases, the Simpson diversity similarly rises. A moderate positive correlation was observed between Shannon and Chao1 (r = 0.69), suggesting that higher species richness is somewhat associated with greater diversity as measured by the Shannon index. In contrast, the correlation between Simpson and Chao1 was weaker (r = 0.43), indicating a lower degree of association between species richness and Simpson diversity compared to the other indices.

**Figure 3:**
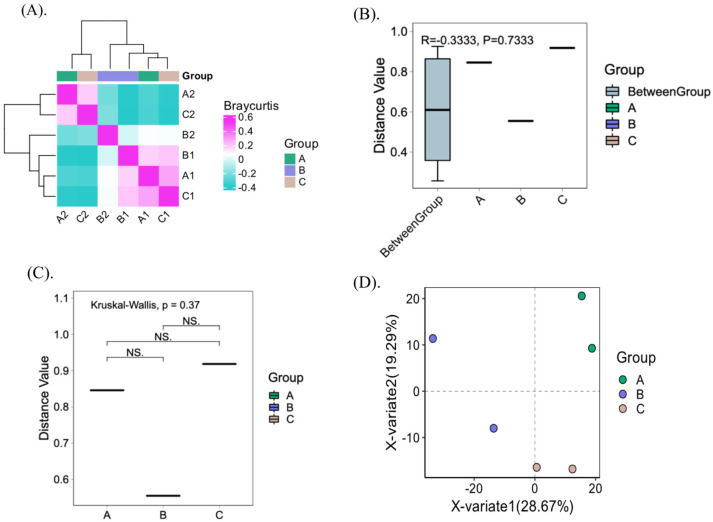
Beta-diversity analysis. (**A**) Sample similarity heatmap based on Bray–Curtis analysis. Both rows and columns represent samples, and the color of the squares represents the similarity between two samples. The left and top show the sample clustering tree, where closer branch distances indicate greater similarity between samples. (**B**) ANOSIM results. (**C**) Statistical differences results. (**D**) PLSDA results. Legend: group A: *C. arabica* L. var. Typica; group B: *C. arabica* L. var. Yellow Caturra; group C: *C. arabica* L. var. Red Caturra; NS: no significant differences.

The shift from unripe (green) to ripe (red/yellow) cherries during fermentation resulted in significant changes in bacterial community composition. Notably, ripe cherries exhibited a significant decrease in pH by day 9 of fermentation (*p* < 0.05), whereas unripe cherries showed minimal pH changes. This acidification aligns with earlier findings and is attributed to microbial metabolic activity during fermentation (Table S3). In addition, at the time of harvest, the total soluble solids (TSSs) values of green and red/yellow cherries differ significantly. This implies that certain species of bacteria may become more prevalent, and others decline because of their ability to survive under acidic conditions during fermentation. Early studies indicated a drop in the pH of the fermentation water from above 6.0 to 4.0 or lower because of these microbial activities [14]. A study conducted recently on Thai Arabica coffee revealed that, under fermentation, the pH drastically decreases, inducing changes in the microbial abundance [12]. The similarity between samples is characterized by the distance between samples, which is one of the results of Beta diversity analysis. The number of microbes present in each sample as well as the abundance of microbes that are shared between them were examined by the Bray–Curtis dissimilarity heatmap (Figure 3A). As observed, group B, Yellow Caturra, was distinct from the Red groups, suggesting that the fruit color (pigmentation) may play a significant role in shaping microbial communities. Red cherries, rich in anthocyanins, may exhibit antimicrobial effects, while yellow cherries, dominated by carotenoids, could foster distinct microbial taxa [46]. Similar patterns in berries suggest that studying coffee cherries’ microbial profiles could reveal deeper links between pigmentation and microbial ecology [46]. Moreover, the heatmap reflects changes in the abundance of microorganisms within the community using color differences. It also displays the similarity between samples and species through clustering. However, the samples are grouped based on their ripe stage (green and yellow/red). Moreover, ANOSIM results showed a uniform distribution between the groups (R values close to “0” (−0.33)) (Figure 3B). The Kruskal–Wallis statistical analysis showed dissimilarities between the groups (Figure 3C). In addition, PLSDA analysis showed a clear separation between the samples at the genus level (Figure 3D). Variable X variate 1 (Axis 1) explained 28.67% of the total variance by loading the samples from the variety Typica (group A) and Red Caturra (group B) in the positive direction, while variable X variate 2 (Axis 2) explained 19.29% by loading the samples from group C (Yellow Caturra) in the negative direction. Understanding these dynamics can inform strategies to control fermentation processes and enhance coffee quality, particularly by fostering beneficial microbial populations. Further investigation into the functional roles of specific microbial taxa and their metabolites could deepen insights into how these changes impact coffee flavor and aroma profiles.

### 3.3. Taxonomic Annotation

The variation in microbial community abundance is a key focus of the study. Table 2 lists the number of species annotated at different taxonomic levels.

**Figure 4:**
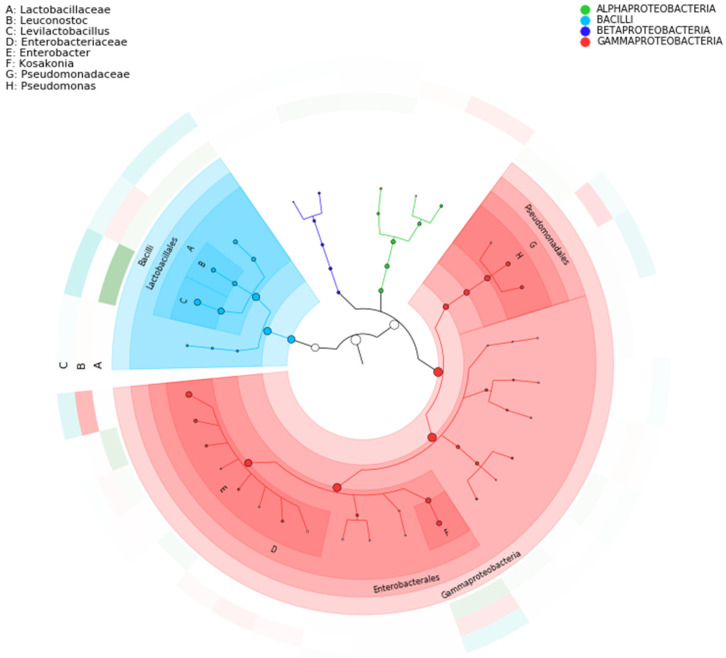
Taxonomical composition and phylogenetic tree of the *C. arabica:* group A: *C.arabica* L. var. Typica; group B: *C. arabica* L. var. Yellow Caturra; group C: *C. arabica* L. var. Red Caturra generated by Graphlan. The colored nodes from the inner to the outer circles represent the top 20 abundant taxa from the phylum to genus level, which are signified by the letters arranged from the outer to the inner circles.

Approximately 99% of the annotated genes were identified as prokaryotic (bacteria and archaea), 0.16% viruses, and 0.05% eukaryotic. At the kingdom level, the main microorganisms category and abundance were 99.78% bacteria, 0.16% viruses, 0.054% eukaryotes, and 0.002% archaea (Table S4). Moreover, the stacked bar plot was used to visualize the relative abundance of different species at various taxonomic levels in different samples/groups. It provides an intuitive view of the dominant species within a community. At the phylum level, Pseudomonadota and Bacillota were the most abundant among the groups/samples (Figure S1). According to the results, groups A and C showed high abundance in Bacillota with a relative abundance of 40.11% and 42.89%, respectively, while group B showed 8.82%. Group B showed high relative abundance in Pseudomonadota (90.68%) (Figure S1). At the Class level, Gammaproteobacteria were predominant in Group B, while Bacilli were predominant in Groups A and C. The difference in distribution between the groups is shown in Figure 4. Although this research lacks multiple sequencing replicates for each sample, the study still provides valuable insights into microbial diversity and functional traits. Furthermore, complementary microbiological analyses across various fermentation batches, assessing microbial prevalence by quantifying specific microorganisms, including LAB, yeasts, Enterobacteriaceae, *E. coli*, and *Staphylococcus*, indicated distinct differences between the three coffee varieties, with yellow cherries exhibiting a higher abundance of pathogens and a lower presence of yeasts and LAB during fermentation. The consistency of these results across replicates suggests that the fermentation process followed a stable microbial pattern, even though full sequencing or additional molecular analyses were not performed on each replicate. The large dataset generated through shotgun metagenomics allows for a comprehensive assessment of microbial composition, functional pathways, and potential implications for coffee quality and safety. Future studies incorporating biological replicates could further strengthen these observations and enhance reproducibility.

**Figure 5:**
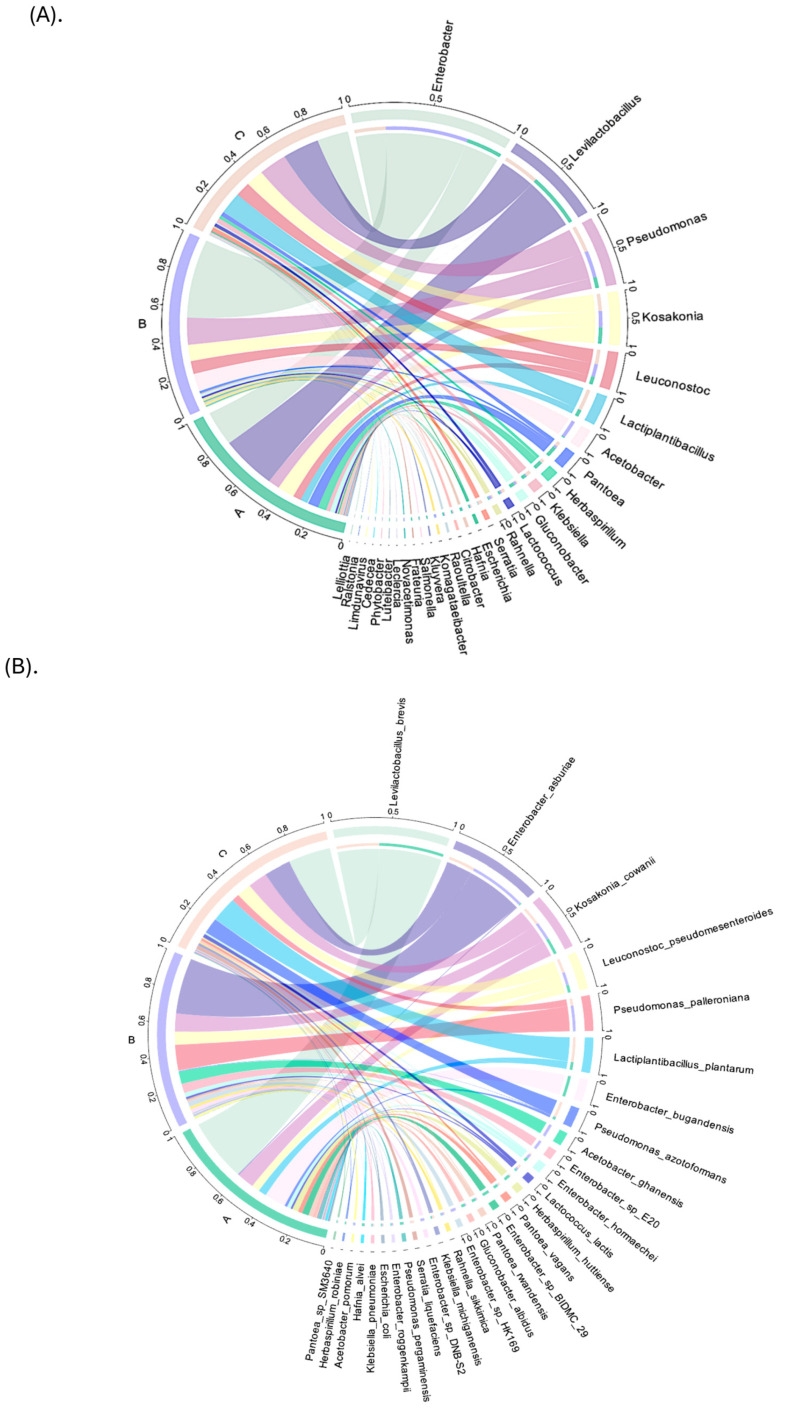
Circus plots revealing the different bacteria at (**A**) genus, and (**B**) species levels among the groups. Legend: Group A: *C.arabica* L. var. Typica; Group B: *C. arabica* L. var. Yellow Caturra; Group C: *C. arabica* L. var. Red Caturra.

Moreover, at the genus level, the samples of the Yellow Caturra (Group B) variety were distinct in the taxa composition of samples from Red Caturra (Group C) and Typica (Group A) varieties (Figure 5A).

*Levilactobacillus* (40–61.2%) and *Lactiplantibacillus* (4–25%) were the dominant genera in Groups A and C, whereas Acetobacter (27%) was notably abundant in Group B. Additionally, *Enterobacter* and *Pseudomonas* were the predominant genera in the green fruits of Group A (sample A1) and Group C (sample C1), while *Enterobacter* was the most prevalent in green fruits of Group B (sample B1) (Figure S2A). This suggests that the microbial composition is potentially related to fruit maturity, color, and genotype and a lesser extent to the variety. Our results agree with early studies indicating the presence of *Enterobacteriaceae* in freshly harvested cherries and *Acetobacteraceae* in fermented cherries of the Typica variety [47]. In a recent study, *Enterobacter* sp. and *Pseudomonas* sp. were found as the most abundant taxa in coffee fermentation [12]. In the current study, *Enterobacter* was the most abundant genera in Yellow Caturra, followed by Typica and Red Caturra (Figure 5A and Figure S2A), while *Acetobacter* was detected in Yellow Caturra only. In addition, *Leviplantibacillus brevis* followed by *Leuconostoc pseudomesenteroides* and *L. plantarum* were the most abundant species in fermented red/yellow cherries (Groups A and C); their abundance varies with the ripe stage and variety. *Enterobacter asburiae* was the most abundant species in Group B. Figure 5B displays the top 30 species with the highest abundance in each group. In addition, the heatmap reflects changes in the abundance of different microorganisms within the community (Figure S2B).

It also displays the similarity between samples and species through clustering. This indicates that *L. brevis* (61% and 40%) and *L. plantarum* (3.6% and 24%) were the most abundant species in the samples A2 and C2 (Typica and Red Caturra cherries), while *E. asburiae* (14.75%)*, Acetobacter ghanensis* (12.4%), and *Pseudomonas palleroniana* (6%) were the most abundant taxa in the B2 sample (Yellow Caturra). However, *L. brevis* was detected for the first time in coffee red/yellow cherries from Caturra but not Typica; we suggest this might be related to the variety. In other studies, *Leuconostoc* was detected as the most prevalent group in coffee fermentation [48], but there is variation in their abundance in fresh arabica coffee cherries [49]. These variations are expected, as bacterial colonization and succession are dynamic processes that can be influenced by a wide range of factors, including local environmental conditions and cherry maturity [49]. Nonetheless, the geographical location and altitude (1400 m above sea level) of the coffee plantation might explain these divergences in the species abundance. Besides, in previous metagenomic study in the Typica variety showed that freshly harvested fruits encompassed bacterial operational taxonomic units belonging to the *Enterobacteriaceae* (i.e., *Klebsiella pneumoniae*), *Gluconobacter* spp., and soil-associated bacteria such as *Dyella kyungheensis*, while wet coffee (de-pulped beans) showed high abundance in *Leuconostoc* [47]. Likewise, early studies in coffee cherries from Taiwan indicate that the most prevalent LABs were *Leuconostoc* sp., *Lactococcus lactis, Enterococcus* sp., and *Weissella* sp. [8]. Moreover, *P. palleroniana* is a plant pathogen causing potato soft rot [50]. At this point, we do not know how this species impacts the coffee quality. Interestingly, *A. ghanensis* was first isolated from traditional heap fermentations of Ghanaian cocoa [51]. These might explain the cacao flavor of the coffee beans, but further studies are required to prove this statement. Overall, the results from our study indicate an abundance of beneficial microorganisms such as LABs (i.e *Levilactobacillus, Lactobacillus*) in fermented red/yellow cherries, while the green cherries are characterized by the abundance of *Enterobacteriaceae* (*Enterobacter, Pseudomonas,* and *Kosakonia*), species. The production of lactic acid and other metabolites during the fermentation process might play a significant role in the final coffee flavor [11]. In addition, in the fermentation process, the LAB strains may generate inhibitory compounds such as bacteriocins which may act as foodborne fungal antagonists; this might explain the low abundance of fungi in the fermented juice. These results demonstrate the great diversity of coffee cherry bacteria, most of which may originate from the environment and some of which may be advantageous or detrimental to the quality and safety of coffee.

### 3.4. Gene Prediction and Functional Analysis

**Figure 6:**
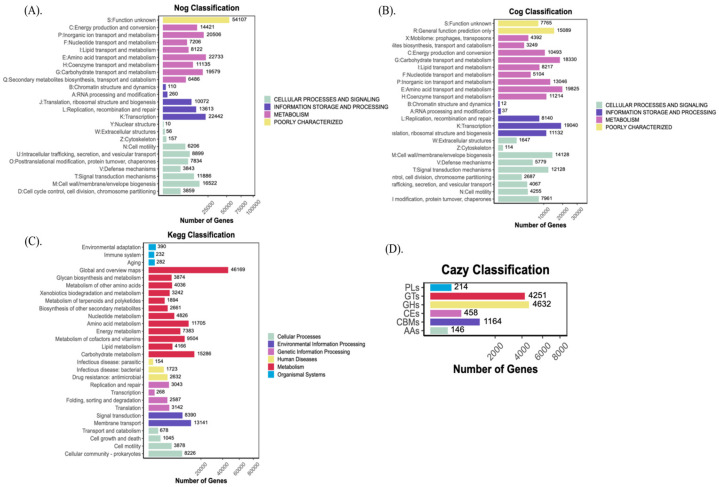
Gene number, distribution and functional group classification according to (**A**) EggNOG; (**B**) COG; (**C**) KEEG, and (**D**) CAZy databases.

Natural coffee processing involves the presence of microorganisms that use the different compounds in the pulp and mucilage as nutrients during the fermentation process. They release other metabolites and organic acids that could influence the beverage’s final sensory qualities [52]. The intricate microbial activity yields ethanol, lactic acid, and a variety of smaller compounds including esters, higher alcohols, aldehydes, and ketones that may permeate into the beans and influence the final makeup of the coffee beverage [45]. Coffee aroma, flavor, and bioactive compounds are enhanced by the metabolites produced by microbes that survive in the coffee cherries [12]. In this study, a high-quality gene catalog was obtained by further removing redundancy based on gene prediction results (Table S5). No statistically significant (*p*-values > 0.05) differences in gene diversity, richness, and dominance indexes between the groups were observed based on the Kruskal–Wallis analysis (Figure S3). A total of 799,658 protein-coding sequences (CDSs) were predicted based on biological gene structure knowledge or distinct annotation databases. However, 205,937 genes were annotated with EggNOG, 181,723 with COG, 155,220 with KEGG, and 10,473 with CAZy. Figure 6A–D depicts the gene category distribution and classification. In addition, 125,884 genes were annotated with the SwissProt database, a curated protein sequence database that provides a high level of protein annotation (e.g., function, domain structure, post-translational modifications, variants, etc.) with little redundancy [31].

### 3.5. EggNOG and COG Annotation Profile

Based on EggNOG annotation, 42.97% of genes were related to the metabolism pathway, 23.11% to cellular processes and signaling, 21.09% were poorly characterized and 12.82% were related to information storage and processing (Figure 6A). Furthermore, the most abundant functional categories in green cherries (samples, A1, B1, and C1) were related to unknown functions, while genes related to transcription, replication, recombination and repair, amino acid transport and metabolism, carbohydrate metabolism were abundant in red/yellow cherries (Figure S4A). Heatmap analysis indicated that the samples originating from green cherries showed a similar NOG profile creating a cluster that was different from the red/yellow cherries, as can be observed from Bray–Curtis dissimilarities and PLSDA results (Figure S5A,C). The X variable 1 (Axis 1) explained 54.63% of the total variance by loading the samples from the green cherries (A1, B1, and C1) in the positive direction, while the X variable 2 (Axis 2) explained 33.23% by loading the samples from yellow/red cherries (A2, B2, and C2). Furthermore, 52%, 29.20%, 12.65%, and 6.2% of genes related to metabolism, cellular processes and signaling, information storage and processing, and poorly characterized were annotated with COG (Figure 6B). COG heatmap analysis showed a clear difference in functional profile between the green and red/yellow fermented cherries (Figure S4B). The highly abundant COG annotated genes were related to transcription, translation ribosomal structure and biogenesis, amino acid transport and metabolism, carbohydrate transport and metabolism, signal transduction mechanisms, general function only, inorganic ion transport and metabolism, and cell wall membrane biogenesis and were detected in green cherries (A1, B1, and C1). Instead, these functional categories were less abundant in red/yellow cherries (Figure S4B). Similarly, a distinct profile was observed between the samples originating from green cherries and red/yellow cherries based on the Bray–Curtis and PLSDA analysis (Figure S5B,C). These differences in the functional profile of microbes associated with green and red/yellow cherries might be related to carbohydrate usage and bacterial survival in fermentation conditions. Untargeted metabolomic studies on coffee cherries at various ripening stages have revealed that most metabolites either directly influence flavor or serve as precursors by regulating enzymatic activity during their formation [53]. Flavor components include flavonoids, which can contribute to astringency and bitterness; free amino acids, which may enhance umami notes; sugars, which provide sweetness; and organic acids, which impart a sour taste.

### 3.6. KEGG Annotation Profile

KEGG annotation results for green and red/yellow fermented cherries indicated that metabolism-related genes were the most abundant, followed by genes related to environmental information processing, genetic information processing, cellular processes, human diseases, and organismal systems (Figure 6C). Furthermore, the metabolism-related genes were most abundant in green cherry samples (Figure S6A). At level 2, global and overview maps were enriched in green and red/yellow fermented cherries, followed by carbohydrate metabolism (Figure S6B). These metabolites were significantly enriched into 210 KEGG pathways, such as 2-oxocarboxylic-acid-metabolism, alanine aspartate and glutamate metabolism, amino sugar and nucleotide sugar metabolism, starch, and sucrose metabolism, fatty acid metabolites, lipid metabolites, etc. These findings suggest that some microbial communities such as lactic acid bacteria are more active and might affect saccharification during vegetable fermentation [54]. These findings could be explained by the fact that *Enterobacter* and *Pseudomonas* were the most prevalent taxa in the green samples, whereas *Levilactobacillus* and *Lactiplantibacillus* were more abundant in the red and yellow cherry samples. Next was amino acid metabolism, which is a precursor to flavor and a major source of nutrients for the growth of microbiota during food fermentation. These may contribute to the overall flavor of the coffee fermentation process. Further KEGG level 3 analysis indicated that transposase, IS30 family (K07482 and K07483), and putative transposase (K07497, K07492) were the most abundant in Red Typica and Red Caturra varieties (Figure S7). The insertion elements encode enzyme transposase required for the excision and insertion of the mobile elements [55]. Further, an iron complex outer membrane receptor protein (K02014), a cold shock protein (K03704), an anti-repressor protein (K07741), and lysozyme (K07273) were abundant in red/yellow cherry samples. These proteins were associated with the presence of predator bacteria from *Gammaproteobacteria* in plants [56]. Moreover, the annotation results showed genes related to glutathione metabolism and fatty acid biosynthesis in all samples (K00799, K00059). Early studies show that glutathione can be synthesized by several microorganisms and plays a crucial role in combating environmental stress by reducing free radicals, and improving cell stress resistance, thus protecting cells from oxidative damage and other chemical stressors [57]. Nonetheless, further studies are required to decipher the role of these metabolites in coffee plants.

### 3.7. CAZy and SwissProt Annotation Profile

In response to shifting environmental conditions (such as the type of dietary substrates available for metabolism), microbial communities can evolve the capacity to produce novel carbohydrate-active enzymes (CAZymes), which are involved in the production of sugar complexes, oligosaccharides, and polysaccharides as well as the breakdown of complex carbohydrates [58]. Of particular interest are glycosyl hydrolases (GHs) and glycosyltransferases (GTs), which can catabolize carbohydrates and are crucial for the taste of fermented foods [59]. In addition, GHs play a critical role in the metabolism to hydrolyze glycosidic bonds, which are composed of carbohydrates and glycosidic portions between carbohydrates [60]. Likewise, GTs play an important role in flavor formation in fermented foods; nonetheless, there are limitations in the impact of microbial CAZyme on coffee flavor. A closer look into the CAZy classification indicates that the most prevalent identified gene clusters were the GHs > GTs > CMBs (carbohydrate-binding module) > CEs (carbohydrate esterase) > and PL (polysaccharide lyase) > AAs (auxiliary activities) families (Figure S8A). Notably, the most abundant categories were genes encoding for GHs and GTs, which can participate in the metabolism and transport of functional and active substances (Figure 6D). The genes were mainly distributed among 10 GTs, 17 GHs, and 2 CMSs families in all samples (Figure S8B). The CMB50 family was the most abundant in red cherries from samples A2 and C2 (Typica and Red Caturra) but not B2 samples which correspond to Yellow Caturra, suggesting that might have something to do with the variety and fruit color. CMB50 are 50 residue modules linked to different enzymes responsible for cleaving peptidoglycan or chitin. Peptidoglycan-targeting enzymes like amidases and peptidases also contain CBM50 modules. These enzymes were found in several bacteria including lactic acid bacteria. Although sample B2 showed a high sugar content and lower pH at the end of fermentation (Table S3), these characteristics do not promote the increase of lactic acid bacteria compared with A2 and C2 samples as observed from the taxonomy annotation. The B2 sample showed a high abundance in *E. asburiae* (14.75%)*, Acetobacter ghanensis* (12.4%), and *Pseudomonas palleroniana* (6%), while A2 and C2 showed a high abundance in *L. brevis* (61% and 40%) and *L. plantarum* (3.6% and 24%). Moreover, the annotation results indicated the most abundant key proteins found in the A2 sample (Red Typica) (Figure S8C). However, LysM domain-containing proteins (AYG30205.1) found in *Lactobacillus*, protein-encoding for tRNA(Ile)-lysidine synthetase (APS42456.1) found in *Weissella*, transposase (ATO44920.1) from *Loigolactobacillus coryniformis,* LysM peptidoglycan-binding domain-containing protein (QOP52666.1) from *Levilactobacillus brevis,* putative minor structural protein (QCZ43716), and lyzozyme M1 (1,4-beta-N-acetylmuramidase) (ATU69464.1) from *L. brevis* were abundant in A2 compared with B2 or C2 metagenome (Figure S8C). These results corroborate the taxonomy results where the *Levilactobacillus, Lactiplantibacillus,* and *Leuconostoc* were the most abundant taxa in A2 samples (Red Typica). It has been documented that *Lactobacillus* and *Leuconostoc* spp. generate lactic acid that promotes yeast growth on the depolymerized media and enzymes such as pectinases and xylanases to depolymerize polysaccharides [61]. In addition, LAB causes metabolite changes in the fermentation process, which includes enhanced fructose consumption and extracellular polysaccharide production [47]. The most prevalent annotated proteins with the SwissProt database are displayed in the heatmap (Figure S9). The two most prevalent protein categories found in sample B1 were P0CB62 and P0DKB3, which stand for the small protein MntS and the protein YmiA, respectively. The function of the membrane protein YmiA, which has been found in *E. coli* and several Gammaproteobacteria, is unknown [62]. Furthermore, *E. coli* K-12 was found to contain the manganese accumulation protein MntS, a small protein that is necessary for bacterial survival in toxic environments [63]. The profiles of samples A1 and C1 were comparable. In addition, the protein profiles of the A2 and C2 samples were similar according to the annotation results, but the B2 sample had a high abundance of P55390 protein, which is likely the cold shock protein y4cH found in *Sinorhizobium fredii* [63]. A closer examination of the taxonomy annotation revealed that the B2 sample’s most prevalent species was *S. fredii*. 

### 3.8. Antibiotic, Biocide, and Metal-Resistant Gene Annotation

**Figure 7:**
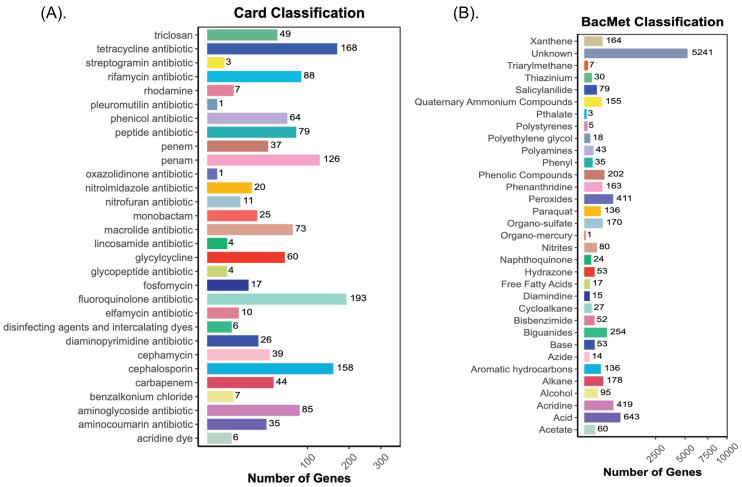
Gene function statistics: (**A**) CARD annotation; (**B**) BacMet annotation, among the groups. The x-axis represents the number of genes, the y-axis represents the functions annotated to genes, and the color of the bars represents groups. The length of the bars represents the abundance.

**Figure 8:**
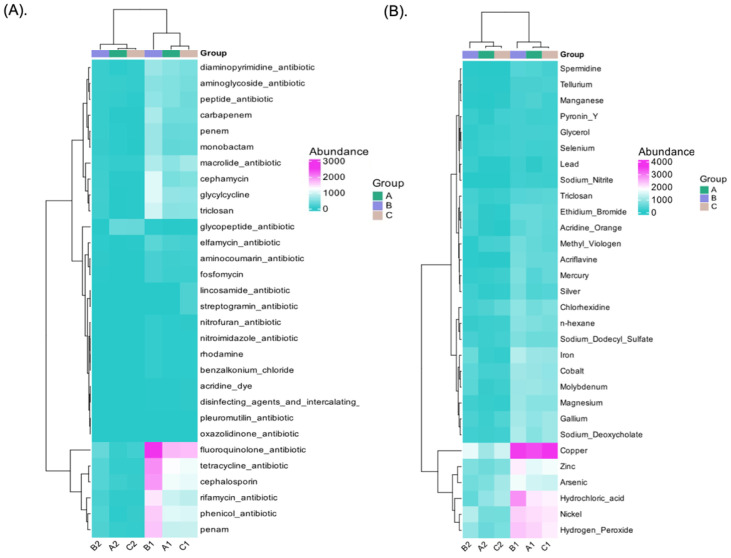
Abundance heatmap across the samples. (**A**) CARD; (**B**) BacMet. Each row represents the annotated function, and each column represents a sample. The color of the squares represents the abundance of genes within the samples. The left side shows the genes clustering tree, and the top side shows the sample clustering tree. Closer branches in the clustering tree indicate greater similarity.

The global threat to human health posed by antibiotic resistance is a persistent and growing problem [64]. The agricultural practices that are widely known to use metal- and biocide-containing products such as zinc, copper, and cadmium in organic and inorganic fertilizers, and pesticides run the risk of providing environments that foster the growth of biocide and metal resistance as well as increased antibiotic resistance as a byproduct of using bactericides [65]. However, is important to comprehend the mechanisms underlying bacterial tolerance to these substances, even in the absence of any possible co-selection for antibiotic resistance. Antibiotic resistance can be co-selected with biocide and metal resistance when certain bacterial species are resistant to both compounds. In this study, 432 ARGs were annotated with CARD, and 8974 BMRGs were annotated with BacMet. Figure 7A,B displays the number of annotated genes and different pathways among the groups. However, the results indicate that the most abundant ARGs were related to fluoroquinolone (11.61%), cephalosporine (10.92%), penam (8.71%), rifamycin (6.08%), aminoglycoside (5.87%), peptide (5.46%), and macrolide (5.04%) antibiotics. Likewise, across the samples, sample B1 originating from green cherry Yellow Caturra showed a high abundance of ARGs associated with these antibiotics (Figure 8A). In addition, cephamycin, glycylcycline, and triclosan antibiotics were abundant in B1, whereas samples A1 and C1 exhibited less abundance of ARGs related to tetracycline, cephalosporin, rifamycin, and penam. The ARG profile depends on the relative abundance of certain bacterial species present in the sample [63]. Despite the lack of a direct correlation between species abundance and antibiotic profile, we can observe from taxonomy annotation that sample B1 had the highest *Enterobacter* abundance when compared to samples A1 and C1.

Bray–Curtis dissimilarities analysis indicated a clear separation between the samples obtained from green cherries vs. yellow/red cherries (Figure S10A,C). Furthermore, among 33 BMRG compounds the most abundant genes were related to an unknown pathway (58.34%), acids (7.15%), acridine (4.66%), peroxides (4.57%), biguanides (2.82%), and phenolic compounds (2.25%). The green cherries (A1, B1, C1) exhibited a markedly increased abundance of BMRGs that provide resistance to a variety of substances, including hydrochloric acid, copper, nickel, hydroxide peroxide, arsenic, and zinc (Figure 8B). Furthermore, among red/yellow cherries higher BMRG abundance similarities were observed between A2 and C2 which correspond to red cherries, while B2 showed a divergent profile (Figure 8B). Bray–Curtis dissimilarities analysis and PLSDA indicated a clear separation between the samples obtained from green cherries vs. yellow/red cherries (Figure S10B,D). Although metals like copper and zinc are vital nutrients that support a variety of cellular and physiological processes in microorganisms, high concentrations of these elements can be toxic [17]. However, while the samples exhibiting zinc resistance displayed tetracycline resistance genes, the green cherries exhibiting a high abundance of genes linked to copper resistance were also resistant to fluoroquinolone. This pattern suggests that there may be a correlation between the abundance profiles of BMRG and ARG in different microbial phyla. Nonetheless, the high levels of *Pseudomonas* and *Enterobacter* in the green cherries could be responsible for the BMRGs and ARGs resistance. The selection of ARGs by different metals, such as those present in animal manure [66] and copper-tailing dam areas [18] has been documented. However, this high prevalence of ARGs and BMRGs suggests that the microbial communities in immature cherries may include organisms that have adapted to resist external stressors, including environmental or agricultural antibiotic residues [67]. These resistance mechanisms help microbes survive in challenging environments and could influence their role during fermentation.

### 3.9. Physicochemical Properties and Microbiome Diversity

The physicochemical parameters (pH, TTA, and TSS) of cherries, ripeness, and the fermentation process can affect their microbiota. The registered parameters are shown in Table S3. A statistically significant difference (*p* < 0.05) in both pH and TSS was found between the green and red/yellow cherries. Samples A1 and C1 (green cherries) from the Typica and Red Caturra varieties displayed the lowest TSS when compared to samples B1 from the Yellow Caturra variety. Comparable TSS values were observed in red/yellow cherries with the highest values in yellow cherries (B2). Likewise, green cherries (samples A1 and C1) showed a slight increase in pH during fermentation, whereas the pH of B1 samples from Yellow Caturra had a significant pH drop. Nonetheless, the pH of the red and yellow fermented cherries decreased significantly due to the production of lactic acid. The correlation analysis (Pearson) reveals key relationships between physicochemical properties and microbial diversity during coffee cherry fermentation (Table 3). A strong negative correlation between TSS and the pH of fermented cherries (−0.942) suggests that higher sugar content leads to a greater drop in pH, likely due to increased microbial activity producing organic acids. Additionally, the positive correlation between acidity and TSS (0.756) indicates that riper cherries, which contain more sugars, tend to exhibit higher acidity. Interestingly, Shannon diversity positively correlates with the pH of fermented cherries (0.623), suggesting that greater microbial diversity may contribute to a more stable pH environment during fermentation. Conversely, the negative correlation between TSS and microbial diversity (−0.435) implies that a higher sugar content may promote specific microbial taxa while limiting overall community diversity. These findings highlight the intricate interplay between fruit ripeness, fermentation dynamics, and microbial ecology, emphasizing the importance of managing these factors to optimize coffee fermentation outcomes.

These findings align with the taxonomy annotation, showing a higher presence of LAB in mature red and yellow cherries (samples A2, B2, C2), while green cherries were more associated with *Enterobacter* species. Notably, despite mature Yellow Caturra cherries (B2) having the highest TSS, this did not correspond to an increase in LAB species. Taxonomic analysis identified *E. asburiae* and *L. pseudomesenteroides* as dominant in B2, whereas *L. brevis, L. plantarum, L. pseudomesenteroides*, and other LAB species were more abundant in A2 and C2. According to an early study on the microbial diversity associated with naturally fermented mature coffee cherries, *E. asburiae* and *L. mesenteroides* were the most common bacterial species from *C. arabica* L. var. *Acaiá*, savannah region (Cerrado) of Brazil [52]. Thus, we appreciate that the bacterial colonization, abundance, and appearance of different species are related to the physicochemical properties of fruits, the ripe stage, color, and the fermentation process.

## 4. Conclusions

This is the first report comparing the microbiome diversity and gene functional profiles of fermented cherries from three varieties of Arabica coffee cultivated in the Intag Valley, Ecuador. It also provides a comprehensive framework for understanding the microbiological biodiversity associated with coffee cherries at different ripe stages, highlighting microbial genes that interact with the fruit and contribute to metabolic functions, safety, and flavor, among other factors. The microbiome analysis indicates a high prevalence of *Leviplantibacillus brevis* in red cherries for the first time. On the other hand, the yellow cherries showed divergent microbiome profiles with *Enterobacter asburiae, Acetobacter ghanensis*, and *Pseudomonas palleroniana*; these microorganisms might be related to the increased BMRGs and ARGs resistance. The detected microorganisms are primarily linked with the cherries’ ripe stage, variety and color, suggesting that the natural fermentation process of coffee gives rise to microbial-niches-specific and specific metabolic pathways. This approach enhances our understanding of coffee fruit microbiota and offers a novel framework for future research aimed at controlling cherry quality and safety during ripening and fermentation. Further studies should focus on identifying potential biological markers linked to Ecuadorian coffees.

## Figures and Tables

**Table 1:** Summary of Assembled Raw Data and Quality Control Results.

Coffee Variety/Group Annotation	Cherries Ripe Stage	Sample	Contig Number	Assembly Length (bp)	N50 (bp)	N90 (bp)	Max Length (bp)	Min Length (bp)	Average Length (bp)
*C. arabica* L. var. Typica/group A	immature green	A1	66,231	138,086,378	9238	621	981,927	300	2084
	mature red	A2	53,051	100,746,497	7152	590	399,572	300	1899
*C. arabica* L. var. Yellow Caturra/group B	immature green	B1	28,951	69,111,399	6264	855	825,737	300	2387
	mature yellow	B2	62,057	90,049,394	3197	485	896,691	300	1451
*C. arabica* L. var. Red Caturra/group C	immature green	C1	29,237	104,006,429	21,010	1197	1,033,477	300	3557
	mature red	C2	61,270	83,508,992	4249	411	723,331	300	1362

**Table 2:** Species annotation counts at different taxonomic levels.

Coffee Variety/Group	Cherries Ripe	Sample	Kingdom	Phylum	Class	Order	Family	Genus	Species
*C. arabica* L. var. Typica/group A	immature green	A1	4	31	62	139	301	921	3407
	immature red	A2	4	32	79	161	341	982	2910
*C. arabica* L. var. Yellow Caturra/group B	immature green	B1	4	32	56	110	227	591	2097
	immature yellow	B2	4	39	76	157	324	902	2855
*C. arabica* L. var. Red Caturra/group C	immature green	C1	4	32	67	145	296	936	3550
	immature red	C2	4	25	45	92	198	555	1870

**Table 3:** Pearson correlation between the physicochemical parameters and microbiome diversity.

Parameter	TSS	pH Cherries Juice	Acidity Cherries Juice (%)	pH Fermented Cherries	Shannon Diversity
TSS	1	−0.216	0.756	−0.942	−0.435
pH Cherries Juice	−0.216	1	0.171	0.082	−0.183
Acidity Cherries Juice (%)	0.756	0.171	1	−0.853	−0.366
pH Fermented Cherries	−0.942	0.082	−0.853	1	0.623
Shannon Diversity	−0.435	−0.183	−0.366	0.623	1

## Data Availability

The raw sequencing data from this research have been submitted to the National Center for Biotechnology Information (NCBI) and are available under accession number PRJNA1207777. The associated BioProject, SUB14995607, was made publicly accessible on 8 January 2025, at https://www.ncbi.nlm.nih.gov/sra/PRJNA1207777.

## Supplementary Materials

The following supporting information can be downloaded at: https://www.mdpi.com/article/10.3390/foods14040614/s1, Table S1. Filtered Raw Data and Quality Control Result Summary; Table S2. Shannon, Simpson and Chao1 indexes; Table S3. Physicochemical parameters of coffee cherries; Table S4. Microorganisms category and relative abundance distribution per sample; Table S5. Table of Gene Prediction and De-duplication Results; Figure S1. Phylum level composition among the group (A) and samples (B); Figure S2. Abundance Heatmap at genus (A) and species (B) levels; Figure S3. Kruskal-Wallis statistical analysis of genes based on different indexes; Figure S4. Abundance heatmap of annotated gene categories with EggNOG (A) and COG (B); Figure S5. Bray-Curtis dissimilarities analysis based on EggNOG (A) and COG (B) gene annotation. Figure S6. Abundance heatmap of annotated gene categories with KEGG (A) and KEGG-level 2 (B). Figure S7. Circus plots revealing the different KO annotation; Figure S8. Abundance heatmap of annotated gene categories with CAZyme (A), CAZyme level 2 (B) and CAZyme level 3 (C). Figure S9. Abundance heatmap of annotated gene categories with SwissProt. Figure S10. Bray-Curtis dissimilarities analysis based on CARD (A) and BacMet (B) gene annotation.

## References

[1] Cheng B., Furtado A., Henry R.J. (2018). The coffee bean transcriptome explains the accumulation of the major bean components through ripening. Sci. Rep..

[2] Mihai R.A., Ortiz-Pillajo D.C., Iturralde-Proaño K.M., Vinueza-Pullotasig M.Y., Sisa-Tolagasí L.A., Villares-Ledesma M.L., Melo-Heras E.J., Cubi-Insuaste N.S., Catana R.D. (2024). Comprehensive Assessment of Coffee Varieties (*Coffea arabica* L. *Coffea canephora* L.) from Coastal, Andean, and Amazonian Regions of Ecuador; A Holistic Evaluation of Metabolism, Antioxidant Capacity and Sensory Attributes. Horticulturae.

[3] De Beenhouwer M., Aerts R., Honnay O. (2013). A global meta-analysis of the biodiversity and ecosystem service benefits of coffee and cacao agroforestry. Agric. Ecosyst. Environ..

[4] (2024). The Cuenca Dispatch. https://thecuencadispatch.com/ecuadorian-coffee-production-is-in-decline-and-now-supplies-only-50-of-national-consumption/#google_vignette.

[5] Torres Castillo N.E., Melchor Martínez E.M., Ochoa Sierra J.S., Ramirez-Mendoza R.A., Parra Saldívar R., Iqba H.M.N. (2020). Impact of climate change and early development of coffee rust: An overview of control strategies to preserve organic cultivars in Mexico. Sci. Total Environ..

[6] Venegas Sánchez S., Orellana Bueno D., Pérez Jara P. (2018). La realidad ecuatoriana en la producción de café. RECIMUNDO.

[7] Cheng B., Furtado A., Smyth H.E., Henry R.J. (2016). Influence of genotype and environment on coffee quality. Trends Food Sci. Technol..

[8] Leong K.H., Chen Y.S., Pan S.F., Chen J.J., Wu H.C., Chang Y.C., Yanagida F. (2014). Diversity of lactic acid bacteria associated with fresh coffee cherries in Taiwan. Curr. Microbiol..

[9] Ivamoto S.T., Reis O., Domingues D.S., Dos Santos T.B., de Oliveira F.F., Pot D., Leroy T., Vieira L.G., Carazzolle M.F., Pereira G.A. (2017). Transcriptome analysis of leaves, flowers and fruits perisperm of *Coffea arabica* L. reveals the differential expression of genes involved in raffinose biosynthesis. PLoS ONE.

[10] De Melo Pereira G.V., de Carvalho Neto D.P., Magalhães Júnior A.I., Vásquez Z.S., Medeiros A.B.P., Vandenberghe L.P.S., Soccol C.R. (2019). Exploring the impacts of postharvest processing on the aroma formation of coffee beans—A review. Food Chem..

[11] Krajangsang S., Seephin P., Tantayotai P., Mahingsapun R., Meeampun Y., Panyachanakul T., Samosorn S., Dolsophon K., Jiamjariyatam R., Lorliam W. (2022). New approach for screening of microorganisms from Arabica coffee processing for their ability to improve *Arabica coffee* flavor. 3 Biotech.

[12] Todhanakasem T., Van Tai N., Pornpukdeewattana S., Charoenrat T., Young B.M., Wattanachaisaereekul S. (2024). The relationship between microbial communities in coffee fermentation and aroma with metabolite attributes of finished products. Foods.

[13] Nam N.N., Do H.D.K., Loan Trinh K.T., Lee N.Y. (2023). Metagenomics: An effective approach for exploring microbial diversity and functions. Foods.

[14] Pothakos V., De Vuyst L., Zhang S.J., De Bruyn F., Verce M., Torres J., Callanan M., Moccand C., Weckx S. (2020). Temporal shotgun metagenomics of an Ecuadorian coffee fermentation process highlights the predominance of lactic acid bacteria. Curr. Res. Biotechnol..

[15] Haile M., Kang W.H. (2019). The role of microbes in coffee fermentation and their impact on coffee quality. J. Food Qual..

[16] Abdelfattah A., Freilich S., Bartuv R., Zhimo V.Y., Kumar A., Biasi A., Salim S., Feygenberg O., Burchard E., Dardick C. (2021). Global analysis of the apple fruit microbiome: Are all apples the same?. Environ. Microbiol..

[17] Balcha E.S., Gómez F., Gemeda M.T., Bekele F.B., Abera S., Cavalazzi B., Woldesemayat A.A. (2023). Shotgun metagenomics-guided prediction reveals the metal tolerance and antibiotic resistance of microbes in poly-extreme environments in the Danakil Depression, Afar Region. Antibiotics.

[18] Chen Y., Chen Y., Shi C., Huang Z., Zhang Y., Li S., Li Y., Ye J., Yu C., Li Z. (2018). SOAPnuke: A mapreduce acceleration-supported software for integrated quality control and preprocessing of high-throughput sequencing data. GigaScience.

[19] Langmead B., Salzberg S.L. (2012). Fast Gapped-Read Alignment with Bowtie 2. Nat. Methods.

[20] Li D., Liu C.M., Luo R., Sadakane K., Lam T.W. (2015). MEGAHIT: An ultra-fast single-node solution for large and complex metagenomics assembly via succinct de Bruijn graph. Bioinformatics.

[21] Zhu W., Lomsadze A., Borodovsky M. (2010). Ab initio gene identification in metagenomic sequences. Nucleic Acids Res..

[22] Fu L., Niu B., Zhu Z., Wu S., Li W. (2012). CD-HIT: Accelerated for clustering the next-generation sequencing data. Bioinformatics.

[23] Patro R., Duggal G., Love M.I., Irizarry R.A., Kingsford C. (2017). Salmon provides fast and bias-aware quantification of transcript expression. Nat. Methods.

[24] Buchfink B., Xie C., Huson D.H. (2015). Fast and sensitive protein alignment using DIAMOND. Nat. Methods.

[25] Jia B., Raphenya A.R., Alcock B., Waglechner N., Guo P., Tsang K.K., Lago B.A., Dave B.M., Pereira S., Sharma A.N. (2017). CARD 2017: Expansion and model-centric curation of the comprehensive antibiotic resistance database. Nucleic Acids Res..

[26] Pal C., Bengtsson-Palme J., Rensing C., Kristiansson E., Larsson D.G. (2014). BacMet: Antibacterial biocide and metal resistance genes database. Nucleic Acids Res..

[27] Huerta-Cepas J., Szklarczyk D., Heller D., Hernández-Plaza A., Forslund S.K., Cook H., Mende D.R., Letunic I., Rattei T., Jensen L.J. (2019). eggNOG 5.0: A hierarchical, functionally and phylogenetically annotated orthology resource based on 5090 organisms and 2502 viruses. Nucleic Acids Res.

[28] Kanehisa M., Goto S. (2000). KEGG: Kyoto encyclopedia of genes and genomes. Nucleic Acids Res..

[29] Galperin M.Y., Makarova K.S., Wolf Y.I., Koonin E.V. (2015). Expanded microbial genome coverage and improved protein family annotation in the COG database. Nucleic Acids Res..

[30] Lombard V., Golaconda Ramulu H., Drula E., Coutinho P.M., Henrissat B. (2014). The carbohydrate-active enzymes database (CAZy) in 2013. Nucleic Acids Res.

[31] Poux S., Arighi C.N., Magrane M., Bateman A., Wei C.H., Lu Z., Boutet E., Bye-A-Jee H., Famiglietti M.L., Roechert B. (2017). On expert curation and scalability: UniProtKB/Swiss-Prot as a case study. Bioinformatics.

[32] Lu J., Rincon N., Wood D.E., Breitwieser F.P., Pockrandt C., Langmead B., Salzberg S.L., Steinegger M. (2022). Metagenome analysis using the Kraken software suite. Nat. Protoc..

[33] Almeida A., Nayfach S., Boland M., Strozzi F., Beracochea M., Shi Z.J., Pollard K.S., Sakharova E., Parks D.H., Hugenholtz P. (2021). A unified catalog of 204,938 reference genomes from the human gut microbiome. Nat. Biotecnol..

[34] Lu J., Breitwieser F.P., Thielen P., Salzberg S.L. (2017). Bracken: Estimating species abundance in metagenomics data. Peer J. Comput. Sci..

[35] Shannon C.E. (1948). A Mathematical Theory of Communication. Bell Syst. Tech. J..

[36] Simpson E. (1949). Measurement of Diversity. Nature.

[37] Chao A. (1984). Non-parametric estimation of the number of classes in a population. Scand. J. Stat..

[38] Tenea G.N., Reyes P. (2024). Bacterial community changes in strawberry fruits (*Fragaria* × *ananassa* variety “Monterey”) from farm field to retail market stands, an indicator of postharvest contamination. Front. Microbiol..

[39] Stat M., Pochon X., Franklin E.C., Bruno J.F., Casey K.S., Selig E.R., Gates R.D. (2013). The distribution of the thermally tolerant symbiont lineage (Symbiodinium clade D) in corals from Hawaii: Correlations with host and the history of ocean thermal stress. Ecol. Evol..

[40] Whittaker R.H. (1960). Vegetation of the Siskiyou Mountains, Oregon and California. Ecol. Monogr..

[41] Anderson M.J., Balakrishnan N., Colton T., Everitt B., Piegorsch W., Ruggeri F., Teugels J.L. (2017). Permutational Multivariate Analysis of Variance (PERMANOVA). Wiley StatsRef: Statistics Reference Online.

[42] Jolliffe I.T., Cadima J. (2016). Principal component analysis: A review and recent developments. Philos. Trans. Ser. A Math. Phys. Eng. Sci..

[43] Xia Y., Sun J., Chen D.G. (2018). Statistical Analysis of Microbiome Data with R.

[44] Association of Official Analytical Chemists (AOAC) (2003). Official Methods of Analysis of AOAC International.

[45] Tang J., Liu Y., Lin B., Zhu H., Jiang W., Yang Q., Chen S. (2021). Effects of ultra-long fermentation time on the microbial community and flavor components of light-flavor Xiaoqu Baijiu based on fermentation tanks. World J. Microbiol. Biotechnol..

[46] Chen C., Zhang Y., Tang W., Chen H., Gong R. (2023). Insights into the Coloring Mechanism of Dark-Red and Yellow Fruits in Sweet Cherry through Transcriptome and Metabolome Analysis. Agronomy.

[47] De Bruyn F., Zhang S.J., Pothakos V., Torres J., Lambot C., Moroni A.V., Callanan M., Sybesma W., Weckx S., De Vuyst L. (2016). Exploring the Impacts of Postharvest Processing on the Microbiota and Metabolite Profiles during Green Coffee Bean Production. Appl. Environ. Microbiol..

[48] De Oliveira Junqueira A.C., de Melo Pereira G.V., Coral Medina J.D., Alvear M.C.R., Rosero R., de Carvalho Neto D.P., Enriquez H.G., Soccol C.R. (2019). First description of bacterial and fungal communities in Colombian coffee beans fermentation analyzed using Illumina-based amplicon sequencing. Sci. Rep..

[49] Mahatmanto T., Sunarharum W.B., Putri F.A., Susanto C.A., Davian A.O., Murdiyatmo U. (2023). The microbiology of arabica and robusta coffee cherries: A comparative study of indigenous bacteria with presumptive impact on coffee quality. FEMS Microbiol. Lett..

[50] Zhang Y., Peng S., Ren Y., Yao T., Chu H., Gao Y., Tian X. (2022). First report of *Pseudomonas palleroniana* causing potato soft rot in China. Plant Dis..

[51] Cleenwerck I., Camu N., Engelbeen K., De Winter T., Vandemeulebroecke K., De Vos P., De Vuyst L. (2007). *Acetobacter ghanensis* sp. nov.; a novel acetic acid bacterium isolated from traditional heap fermentations of Ghanaian cocoa beans. Int. J. System. Evol. Microbiol..

[52] Evangelista S.R., Miguel M.G., Silva C.F., Pinheiro A.C., Schwan R.F. (2015). Microbiological diversity associated with the spontaneous wet method of coffee fermentation. Int. J. Food Microbiol..

[53] Li Z., Zhou B., Zheng T., Zhao C., Shen X., Wang X., Qiu M., Fan J. (2023). Integrating metabolomics and proteomics technologies provides insights into the flavor precursor changes at different maturity stages of Arabica coffee cherries. Foods.

[54] Liang T., Jiang T., Liang Z., Zhang N., Dong B., Wu Q., Gu B. (2023). Carbohydrate-active enzyme profiles of *Lactiplantibacillus plantarum* strain 84-3 contribute to flavor formation in fermented dairy and vegetable products. Food Chem..

[55] Szabó M., Kiss J., Olasz F. (2010). Functional Organization of the Inverted Repeats of IS30. J. Bacteriol..

[56] Sieradzki E.T., Nuccio E.E., Pett-Ridge J., Firestone M.K. (2023). Expression of macromolecular organic nitrogen degrading enzymes identifies potential mediators of soil organic N availability to an annual grass. ISME J..

[57] Xue Z.P., Cu X., Xu K., Peng J.H., Liu H.R., Zhao R.T., Wang Z., Wang T., Xu Z.S. (2023). The effect of glutathione biosynthesis of *Streptococcus thermophilus* ST-1 on cocultured *Lactobacillus delbrueckii* ssp. *bulgaricus* ATCC11842. J. Dairy Sci..

[58] Neves A.L.A., Yu J., Suzuki Y., Baez-Magana M., Arutyunova E., O’Hara E., McAllister T., Ominski K.H., Lemieux M.J., Guan L.L. (2021). Accelerated discovery of novel glycoside hydrolases using targeted functional profiling and selective pressure on the rumen microbiome. Microbiome.

[59] You L., Yang C., Jin H., Kwok L.-Y., Sun Z., Zhang H. (2022). Metagenomic features of traditional fermented milk products. LWT-Food Sci. Technol..

[60] Tingley J.P., Low K.E., Xing X., Abbott D.W. (2021). Combined whole cell wall analysis and streamlined in silico carbohydrate-active enzyme discovery to improve biocatalytic conversion of agricultural crop residues. Biotechnol. Biofuels.

[61] Avallone S., Brillouet J.M., Guyot B., Olguin E., Guiraud J.P. (2002). Involvement of pectolytic micro-organisms in coffee fermentation. Int. J. Food Sci. Technol..

[62] Waters L.S., Sandoval M., Storz G. (2011). The *Escherichia coli* MntR miniregulon includes genes encoding a small protein and an efflux pump required for manganese homeostasis. J. Bacteriol..

[63] O’Connell K.P., Gustafson A.M., Lehmann M.D., Thomashow M.F. (2000). Identification of cold shock gene loci in *Sinorhizobium meliloti* by using a luxAB reporter transposon. Appl. Environ. Microbiol..

[64] Zhang D., Peng Y., Chan C.L., On H., Wai H.K., Shekhawat S.S., Gupta A.B., Varshney A.K., Chuanchuen R., Zhou X. (2021). Metagenomic survey reveals more diverse and abundant antibiotic resistance genes in municipal wastewater than hospital wastewater. Front. Microbiol..

[65] Dean R.J., Shimmield T.M., Black K.D. (2007). Copper, zinc and cadmium in marine cage fish farm sediments: An extensive survey. Environ. Pollut..

[66] Berg J., Tom-Petersen A., Nybroe O. (2005). Copper amendment of agricultural soil selects for bacterial antibiotic resistance in the field. Lett. Appl. Microbiol..

[67] Li J., Ma Q., Jin M., Huang L., Hui D., Sardans J., Peñuelas J., O’Connor P., Zhu Y., Yang X. (2024). From grasslands to genes: Exploring the major microbial drivers of antibiotic-resistance in microhabitats under persistent overgrazing. Microbiome.

