# Supplementary material for: Unveiling the Microbial Signatures of Arabica Coffee Cherries: Insights into Ripeness Specific Diversity, Functional Traits, and Implications for Quality and Safety
Source: Foods. 2025 Feb 12;14(4):614. doi: 10.3390/foods14040614 (PMC11854473; doi:10.3390/foods14040614)

Supplementary Materials

Table S1. Filtered Raw Data and Quality Control Result Summary

Sample name	Raw_Reads	Raw_Bases	Clean_Reads	Clean_Bases	Clean_Data_Rate	Q20	Q30	GC	Clean_Reads_Remove_Host	Clean_Bases_Remove_Host	Host_Rate
Average	69493333	10424000000	66920266	1.0038E+10	96.31	97.16	92.65	51.44	66817469	1.002E+10	0.153587
A1	69200000	10380000000	66842070	1.0026E+10	96.59	97.72	94.99	55.34	66832860	1.002E+10	0.013779
A2	70880000	10632000000	66899198	1.0035E+10	94.38	97.52	92.58	45.57	66602302	9.99E+09	0.443796
B1	69760000	10464000000	66822064	1.0023E+10	95.79	97.03	92.61	55.33	66812280	1.002E+10	0.014642
B2	69120000	10368000000	66978112	1.0047E+10	96.9	97.02	92.38	52.66	66928462	1.004E+10	0.074129
C1	68800000	10320000000	67013562	1.0052E+10	97.4	96.43	91.69	55.93	67011128	1.005E+10	0.003632
C2	69200000	10380000000	66966594	1.0045E+10	96.77	97.26	91.65	43.79	66717782	1.001E+10	0.371546

Sample: Sample Name
Raw Reads: Raw Reads Count
Raw Bases: Raw Bases Count
Clean Reads: Clean Reads Count After QC
Clean Bases: Clean Bases Count After QC
Clean Data Rate: Clean Data Percentage (%)
Q20: Q20 Base Content (%)
Q30: Q30 Base Content (%)
GC: GC Content (%)
Clean Reads (host removed): Clean Reads Count After Host Removal
Clean Bases (host removed): Clean Bases Count After Host Removal
Host Rate: Host Reads Percentage (%).

Table S2. Shannon, Simpson, and Chao1 indexes.

Coffee variety/ Group	Cherries ripe	Sample	Shannon	Simpson	Chao1
C. arabica L. var. Typica / group A	immature green	A1	5.59566708	0.93054987	3407
	mature-red	A2	2.89896639	0.61590728	2910
C. arabica L. var. Yellow Caturra /group B	immature green	B1	3.70877972	0.79114188	2097
	mature-yellow	B2	5.24696641	0.92622942	2855
C. arabica L. var. Red Caturra / group C	immature green	C1	4.90033676	0.8831025	3550
	mature-red	C2	3.03867822	0.75710323	1870

Table S3. Physicochemical parameters of coffee cherries

Coffee variety/ Group	Cherries ripe	Sample	Total soluble solids (TSS) of cherries juice	pH cherries juice	Acidity cherries juice (%)	pH fermented cherries
C.arabica L. var. Typica / group A	immature green	A1	1.72 ± 0.01^{a}	5.53 ± 0.01^{bcA}	0.10 ± 0.01^{a}	5.78 ± 0.01^{dA}
	mature-red	A2	9.33 ± 0.01^{cd}	4.99 ± 0.01^{aA}	0.20 ± 0.01^{ab}	3.85 ± 0.01^{aB}

C. arabica L. var. Yellow Caturra /group B	immature green	B1	8.33 ± 0.01^{c}	5.25 ± 0.01^{bA}	0.12 ± 0.01^{ab}	4.72 ± 0.01^{bB}
	mature-yellow	B2	13.53 ± 0.05^{e}	4.96 ± 0.01^{aA}	0.26 ± 0.01^{b}	3.73 ± 0.01^{aB}
C. arabica L. var. Red Caturra / group C	immature green	C1	3.01 ± 0.01^{b}	5.31 ± 0.01^{bA}	0.17 ± 0.01^{ab}	5.39 ± 0.01^{cdA}
	mature-red	C2	10.73 ± 0.05^{d}	$5.99 \pm 0.01^{c\,A}$	0.29 ± 0.01^{b}	3.62 ± 0.01^{aB}

Data are means ± standard error. Values with different letters are significantly different $P < 0.05$. Small letters show the difference between within the column; Capital letter in the raw show the differences of pH of cheery juice and fermented cherries (Duncan's test).

Table S4. Microorganisms category and relative abundance distribution per sample

**Category**	*C. arabica* L. var. Typica		*C. arabica* L. var. Yellow Caturra		*C. arabica* L. var. Red Caturra	
	immature-green	mature-red	immature-green	mature-yellow	immature-green	mature-red
	A1	A2	B1	B2	C1	C2
Bacteria	99.97%	99.49%	100.00%	99.37%	99.99%	99.87%
Eukaryota	0.01%	0.16%	0.00%	0.10%	0.00%	0.05%
Archaea	0.00%	0.00%	0.00%	0.01%	0.00%	0.00%
Viruses	0.01%	0.36%	0.00%	0.52%	0.00%	0.08%

Table S5. Gene Prediction and De-duplication Results

**Sample**	**Predicted CDS number**	**Removed Dup. CDS number**
A1	187990	131313
A2	134534	89634
B1	90215	66743
B2	136289	85266
C1	121327	93812
C2	129303	76865
Merge Total	799658	324464

Sample: Sample name
Predicted CDS number: Predicted gene total number
Removed. Dup. CDS number: Gene number after de-duplication.

Figure S1. Phylum level composition among the group (A) and samples (B). The x-axis represents samples/groups, the y-axis represents the relative abundance of species, and the color of the bars represents different species. Longer bars indicate higher relative abundance.

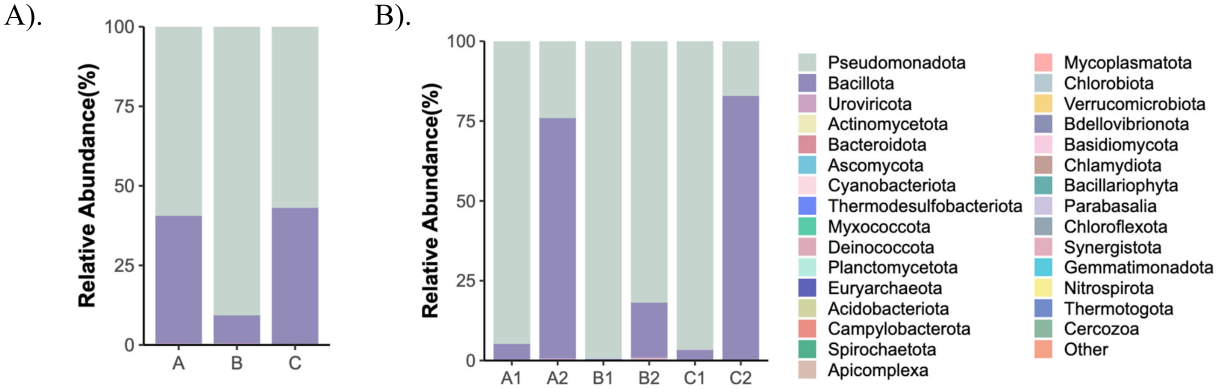

Figure S2. Abundance Heatmap at genus level. Each row represents a taxonomic unit, and each column represents a sample and group. The heatmap displays the top 30 taxonomic units with the highest abundance. The color of the squares represents the abundance of genera within the samples. The left side shows the genera clustering tree, and the top side shows the sample clustering tree. Closer branches in the clustering tree indicate higher similarity.

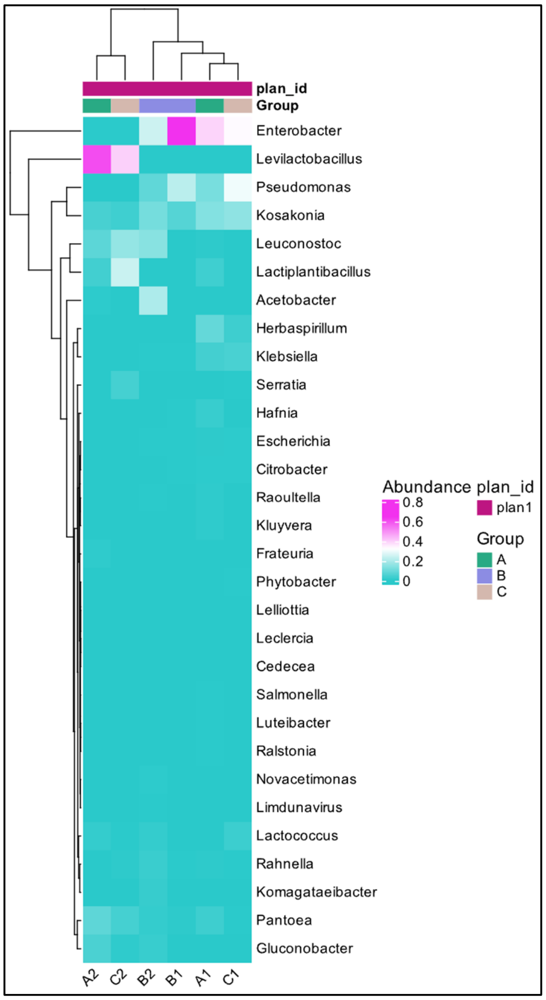

Figure S3. Kruskal-Wallis statistical analysis of genes based on different indexes.

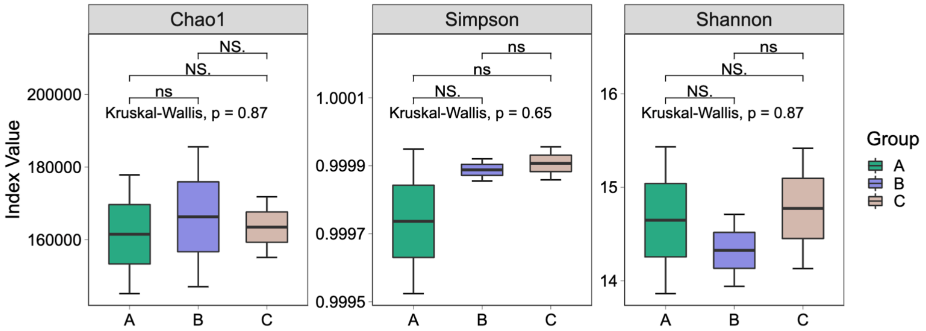

Figure S4. Abundance heatmap of annotated gene categories with EggNOG (A) and COG (B). Each row represents a functional category, and each column represents a sample. The color of the squares represents the abundance of each category within the samples. The left side and top side show the samples clustering tree. Closer branches in the clustering tree indicate higher similarity.

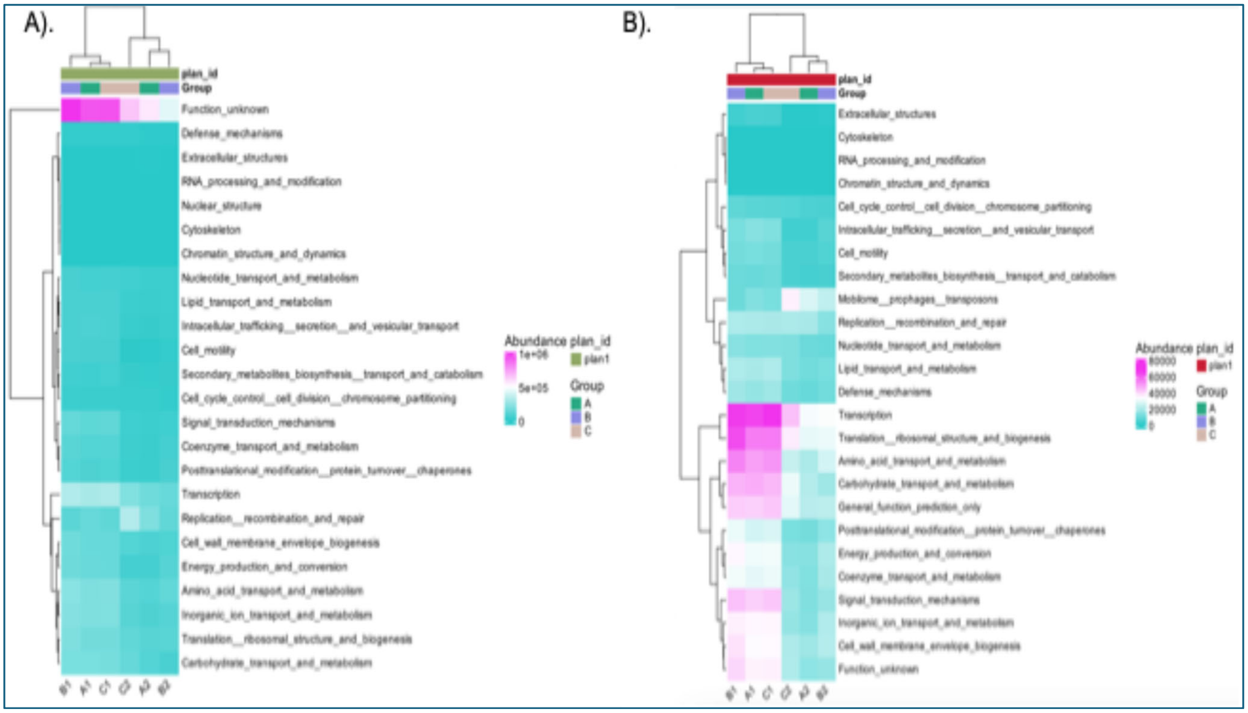

Figure S5. Bray-Curtis dissimilarities analysis based on EggNOG (A) and COG (B) gene annotation. The color of the squares represents the abundance of each category within the samples. The left side and top side show the samples clustering tree. Closer branches in the clustering tree indicate higher similarity. Results of PLSDA analysis based on EggNOG (C) and COG (D) results. Legend: group A: *C.arabica* L. var. Typica; group B: *C. arabica* L. var. Yellow Caturra; group C: *C. arabica* L. var. Red Caturra.

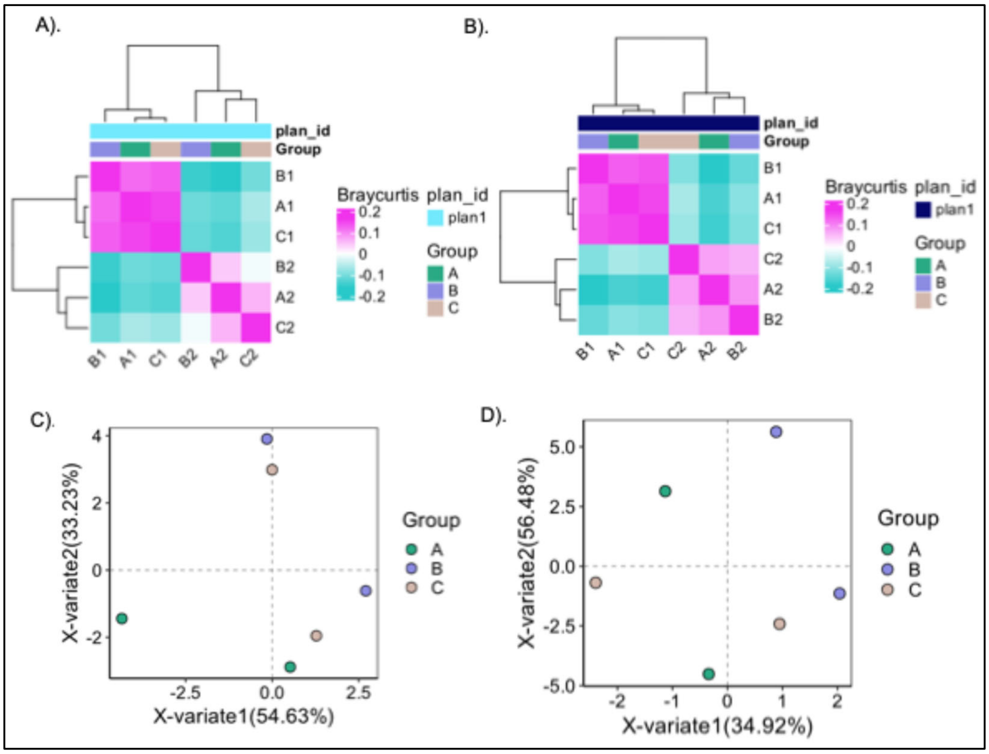

Figure S6. Abundance heatmap of annotated gene categories with KEGG (A) and KEGG-level 2 (B). Each row represents a functional category, and each column represents a sample. The color of the squares represents the abundance of each category within the samples. The left side and top side show the samples clustering tree. Closer branches in the clustering tree indicate higher similarity.

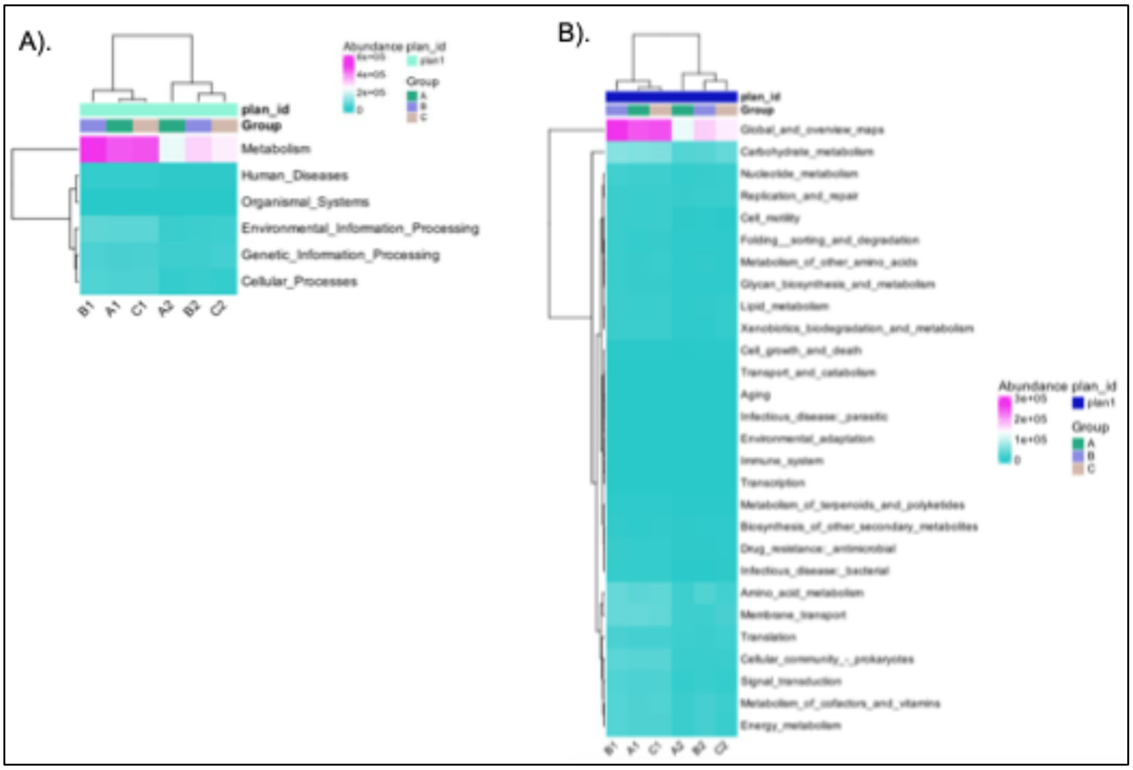

Figure S7. Circus plots revealing the different KO annotation. Legend: group A: *C.arabica* L. var. Typica; group B: *C. arabica* L. var. Yellow Caturra; group C: *C. arabica* L. var. Red Caturra.

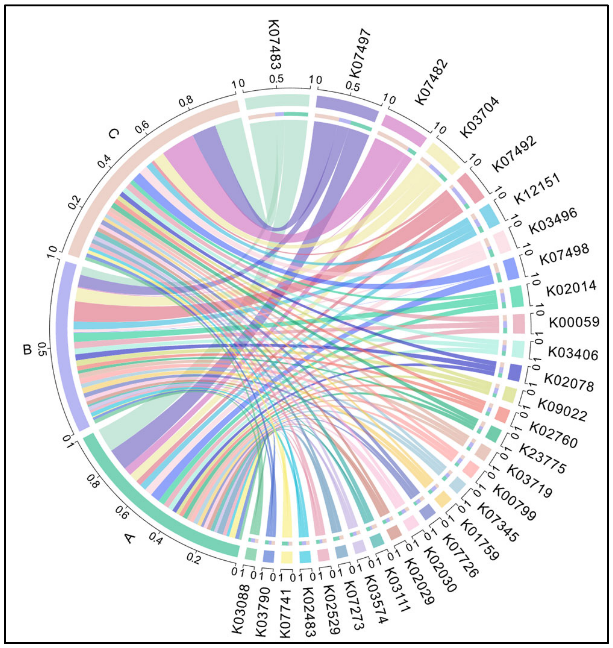

Figure S8. Abundance heatmap of annotated gene categories with CAZyme (A), CAZyme level 2 (B) and CAZyme level 3 (C). Each row represents a functional category, and each column represents a sample. The color of the squares represents the abundance of each category within the samples. The left side and top side show the samples clustering tree. Closer branches in the clustering tree indicate higher similarity.

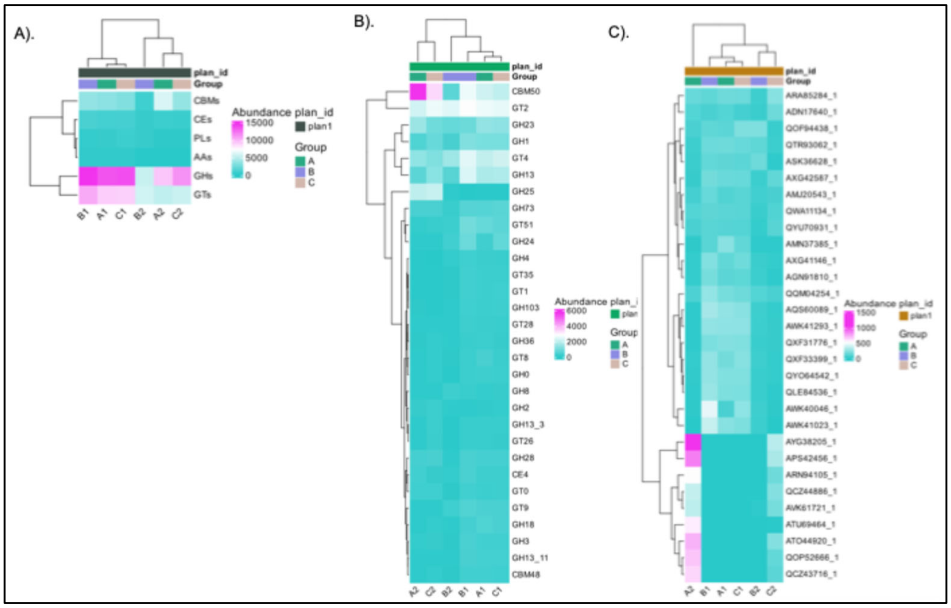

Figure S9. Abundance heatmap of annotated gene categories with SwissProt Each row represents a functional category, and each column represents a sample. The color of the squares represents the abundance of each category within the samples. The left side and top side show the samples clustering tree. Closer branches in the clustering tree indicate higher similarity.

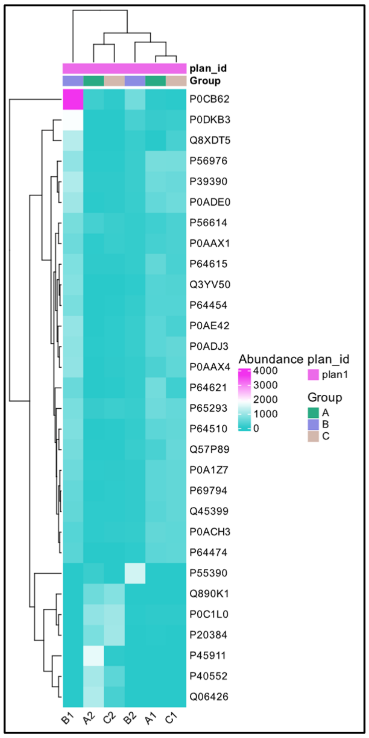

Figure S10. Bray-Curtis dissimilarities analysis based on CARD (A) and BacMet (B) gene annotation. The color of the squares represents the abundance of each category within the samples. The left side and top side show the samples clustering tree. Closer branches in the clustering tree indicate higher similarity. Results of PLSDA analysis based on CARD (C) and BacMet (D) results. Legend: group A: *C.arabica* L. var. Typica; group B: *C. arabica* L. var. Yellow Caturra; group C: *C. arabica* L. var. Red Caturra.

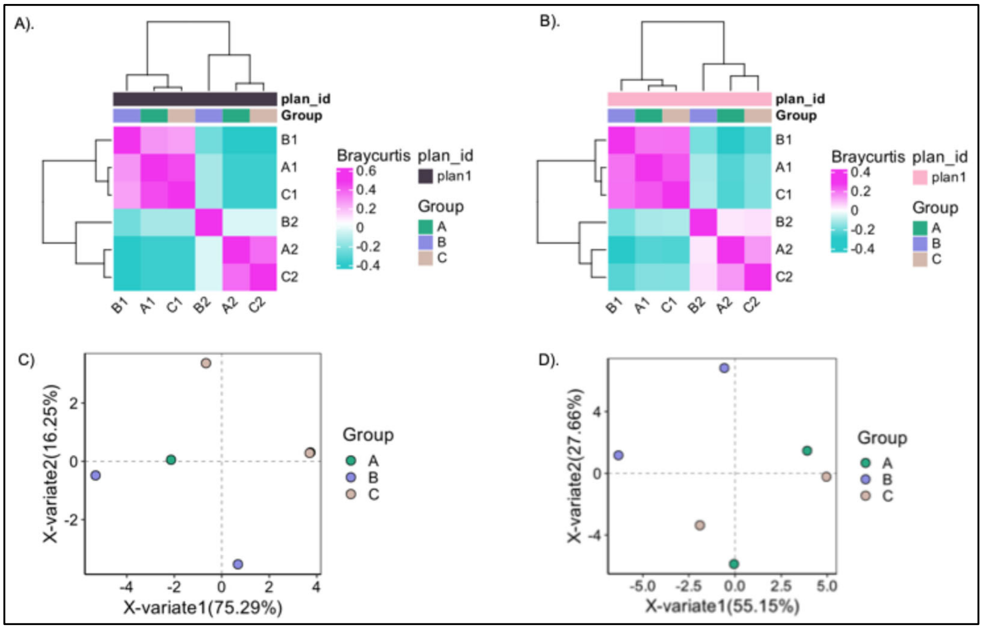

Supplement: Supplementary file 1 [file foods-14-00614-s001.zip › foods-3447717-supplementary.pdf]
